# Evidence that toxin resistance in poison birds and frogs is not rooted in sodium channel mutations and may rely on “toxin sponge” proteins

**DOI:** 10.1085/jgp.202112872

**Published:** 2021-08-05

**Authors:** Fayal Abderemane-Ali, Nathan D. Rossen, Megan E. Kobiela, Robert A. Craig, Catherine E. Garrison, Zhou Chen, Claire M. Colleran, Lauren A. O’Connell, J. Du Bois, John P. Dumbacher, Daniel L. Minor

**Affiliations:** 1 Cardiovascular Research Institute, University of California, San Francisco, San Francisco, CA; 2 School of Biological Sciences, University of Nebraska–Lincoln, Lincoln, NE; 3 Department of Chemistry, Stanford University, Stanford, CA; 4 Department of Biology, Stanford University, Stanford, CA; 5 Institute for Biodiversity Science and Sustainability, California Academy of Sciences, San Francisco, CA; 6 Department of Biology, San Francisco State University, San Francisco, CA; 7 Department of Biochemistry and Biophysics, University of California, San Francisco, San Francisco, CA; 8 Department of Cellular and Molecular Pharmacology, University of California, San Francisco, San Francisco, CA; 9 California Institute for Quantitative Biomedical Research, University of California, San Francisco, San Francisco, CA; 10 Kavli Institute for Fundamental Neuroscience, University of California, San Francisco, San Francisco, CA; 11 Molecular Biophysics and Integrated Bio-imaging Division, Lawrence Berkeley National Laboratory, Berkeley, CA

## Abstract

Many poisonous organisms carry small-molecule toxins that alter voltage-gated sodium channel (Na_V_) function. Among these, batrachotoxin (BTX) from *Pitohui* poison birds and *Phyllobates* poison frogs stands out because of its lethality and unusual effects on Na_V_ function. How these toxin-bearing organisms avoid autointoxication remains poorly understood. In poison frogs, a Na_V_ DIVS6 pore-forming helix N-to-T mutation has been proposed as the BTX resistance mechanism. Here, we show that this variant is absent from *Pitohui* and poison frog Na_V_s, incurs a strong cost compromising channel function, and fails to produce BTX-resistant channels in poison frog Na_V_s. We also show that captivity-raised poison frogs are resistant to two Na_V_-directed toxins, BTX and saxitoxin (STX), even though they bear Na_V_s sensitive to both. Moreover, we demonstrate that the amphibian STX “toxin sponge” protein saxiphilin is able to protect and rescue Na_V_s from block by STX. Taken together, our data contradict the hypothesis that BTX autoresistance is rooted in the DIVS6 N→T mutation, challenge the idea that ion channel mutations are a primary driver of toxin resistance, and suggest the possibility that toxin sequestration mechanisms may be key for protecting poisonous species from the action of small-molecule toxins.

## Teaser

Some poisonous animals produce toxins that affect Na^+^ channel function yet avoid autointoxication. Abderemane-Ali et al. show that resistance by *Pitohui* birds and *Phyllobates* frogs to their own toxins may be mediated not by mutations in their channels, but by toxin-sequestering “sponge” proteins.

## Introduction

Many organisms harbor various small-molecule toxins that target ion channels as a means of defense from predation (Savitzky et al., 2012). Among these, batrachotoxin (BTX), a diet-acquired (Daly et al., 1994b; Daly et al., 1994a; Dumbacher et al., 2004) steroidal amine found in distantly related vertebrate lineages, including poisonous birds (*Pitohui* spp. and *Ifrita kowaldi*; Dumbacher et al., 1992; Dumbacher et al., 2000) and neotropical poison frogs (*Phyllobates*; Santos et al., 2016), stands out because of its lethality and its unusual ability to facilitate opening and prevent inactivation of voltage-gated sodium channels (Na_V_s; Catterall, 1977; Khodorov, 1985; Logan et al., 2016; Wang and Wang, 2003). This lipophilic, steroidal neurotoxin is thought to bind in the Na_V_ inner pore (Wang and Wang, 2003). How vertebrates that bear BTX or other small-molecule toxins avoid autointoxication remains unresolved (Arbuckle et al., 2017; Almabruk et al., 2018; Hunter, 2018). Toxin-resistant mutants of target ion channels in host organisms (Tarvin et al., 2017; Bricelj et al., 2005; Hanifin and Gilly, 2015; Jost et al., 2008) or their predators (Geffeney et al., 2002; Geffeney et al., 2005; McGlothlin et al., 2016) have been suggested as the primary drivers of toxin resistance (Santos et al., 2016), an idea supported by examples of tetrodotoxin (TTX)-resistant (Hanifin and Gilly, 2015; Jost et al., 2008; Geffeney et al., 2002; Geffeney et al., 2005; McGlothlin et al., 2016) and saxitoxin (STX)-resistant (Bricelj et al., 2005) Na_V_s, as well as epibatidine-resistant nicotinic acetylcholine receptors (Tarvin et al., 2017) found in toxin-carrying metazoans. In poison frogs, an Na_V_ domain IV segment 6 (DIVS6) pore-forming helix N→T mutation has been proposed as the BTX resistance mechanism (Tarvin et al., 2016; Wang and Wang, 2017). Although the DIVS6 N→T change reduces BTX sensitivity when tested in rat Na_V_1.4 (Wang and Wang, 2017), this variant occurs with very low frequency among *Phyllobates terribilis* (Márquez et al., 2019), and is absent from *Phyllobates aurotaenia* (Tarvin et al., 2016; Márquez et al., 2019), another poison frog having high BTX levels (Albuquerque et al., 1973). Given these issues and the absence of functional studies of poison frog Na_V_s, whether BTX-bearing animals rely on Na_V_ mutations or other BTX autoresistance mechanisms remains unclear.

Here, we clone and characterize Na_V_s from the BTX-bearing bird *Pitohui uropygialis meridionalis* (*Pum*) and two poison frog species that carry alkaloid toxins in the wild (*P. terribilis* [BTX]; *Dendrobates tinctorius* histrionicotoxin [HTX] and pumiliotoxin [PTX]). We found that the DIVS6 N→T variant is absent from *Pitohui* and poison frog Na_V_s, incurs a strong cost that compromises channel function, and fails to produce BTX-resistant channels when tested in the context of poison frog Na_V_s. Most surprising, poison frogs proved resistant to BTX poisoning and poisoning by another small-molecule toxin, STX, despite expressing Na_V_s that are sensitive to both toxins. We further show that saxiphilin (Sxph), a high-affinity STX-binding protein found in frog plasma and organs (Yen et al., 2019; Mahar et al., 1991; Doyle et al., 1982; Morabito and Moczydlowski, 1994), can protect and rescue Na_V_-expressing cells from STX poisoning by sequestering the toxin. Hence, our data challenge the hypothesis that BTX autoresistance is rooted in Na_V_ mutations, underscore the trade-offs between toxin-resistant mutations and fitness cost (Hague et al., 2018), and highlight the potential importance of alternative mechanisms such as toxin sequestration as strategies for protecting toxin-bearing species from autointoxication and environmental small-molecule threats.

## Materials and methods

### Identification and cloning of *Pitohui* Na_V_1.4, Na_V_1.5, and Na_V_β2

Genomic DNA from *Pum* (family Oriolidae) blood and tissue was extracted using DNeasy kits (Qiagen) to create whole-genome sequence libraries for the poisonous *Pitohui* birds. Tissue samples were collected in 1989 near the village of Bonua, Central Province, Papua New Guinea (10°08′S by 149°10′30″E), stored in ethanol in the field, and frozen since being in the laboratory. Two genome sequence libraries were created using Illumina Nextera kits. One library had a target insert size of 500–640 bp and occupied a full run on the MiSeq genetic analyzer using 300-bp paired-end reads. The second library had a target insert size of 640–709 bp and occupied a full lane of a HiSeq 2500 in rapid run mode using 150-bp paired-end reads.

The MiSeq run returned 16,279,946 paired-end reads. The program BBmerge version 4.0 (US Department of Energy Joint Genome Institute; https://jgi.doe.gov/data-and-tools/bbtools/bb-tools-user-guide/bbmerge-guide/) was used to join the forward and reverse reads into a single long read. 7,050,488 of the read pairs (∼43%) were joined, and the remaining reads were retained as paired-end reads or single reads for later analyses. The average size of merged reads was 542.6 bases. All reads were then trimmed using Trimmomatic (Bolger et al., 2014) for minimum length, removing adapters, and performing basic quality filtering. All unmerged and unpaired reads were combined into a single FastQ file.

The HiSeq run returned 150,979,291 paired-end reads. We removed adapters, trimmed for minimum length, and performed basic quality filtering using Trimmomatic (Bolger et al., 2014). 130,607,604 pairs of reads (86.51%) passed filtering, another 9,199,536 (6.09%) of forward-only reads passed filter, and 3,342,519 (2.21%) of reverse-only reads passed filter. These two read sets (the MiSeq and HiSeq Illumina datasets) composed the data for gene assembly.

*Corvus brachyrhynchos* and *Corvus cornix* crows (family Corvidae; from Joel McGlothlin, Virginia Tech, Blacksburg, VA) were the closest living relatives of *Pitohui*s that had a fully annotated genome in the sodium channel gene regions. Crow sequences were used as reference sequences for BLAST searches of *Pitohui* sequences and for assisting with *Pitohui* Na_V_ assembly. These sequences included the complete nuclear DNA sequence with all exons, introns, and upstream and downstream untranslated regions.

To assemble the SCN4A gene from our *Pitohui* reads, we used the following pipeline, written as a Bash shell script. First, we used BLATq version 1.0.2 (https://github.com/calacademy-research/BLATq), which uses BLAT (Kent, 2002; https://users.soe.ucsc.edu/~kent), to search all Illumina data (those merged and unmerged, as well as paired and unpaired reads) for any sequences that aligned with the full genome sequences of one of the *Corvus* Na_V_1.4 sodium channel genes. We then used the script excerptByIDs version 1.0.2 (https://github.com/calacademy-research/excerptByIDs) to create a new FastQ file consisting of only the Illumina reads with strong BLATq scores. These files were combined into a single set of all BLAT “hits” using the Unix “cat” command. We then used the assembler SPAdes 3.9.0 (Bankevich et al., 2012) to perform an initial de novo assembly of reads in the hit file. We improved upon this assembly by using the genome assembly program PRICE version 1.2 (Ruby et al., 2013), which iteratively extends the assembly beginning with the assembled contigs from SPAdes, and extending using the paired-end Illumina read data (the entire HiSeq paired-end data as well as the unmerged MiSeq data). See Data S1 for the assembly Bash shell script and options and parameters.

The assembled contigs from PRICE were loaded into Geneious (versions 8.0 through 11.0.2). Within Geneious, we used BLAST to identify which contigs contained the *Pitohui* sequences. We created a BLAST database consisting of all of the assembled PRICE contigs, and we used each of the *Corvus* Na_V_1.4 exons to query the BLAST database of contigs using MegaBLAST. The top hits for each exon suggested which assembled contigs contained the Na_V_1.4 sequence, and typically several to all of the exons were found on the same contig. Assuming that the exon splice patterns were identical in crow and *Pitohui*, we aligned each of the crow Na_V_1.4 exon sequences to the top-hit *Pitohui* contig, and we annotated the matching exon regions as the *Pitohui* SCN4A exons.

This assembly and annotation pipelines were repeated for primary Na_V_s Na_V_1.5 α-subunit (SCN5A) and the Na_V_ β-subunit (SCN2B).

### Cloning of poison frog Na_V_s

Skeletal muscle was harvested from captive *P. terribilis* and *D. tinctorius* (Josh’s Frogs) after euthanasia in accordance with University of California, San Francisco Institutional Animal Care and Use Committee (UCSF IACUC) protocol AN136799. Total RNA and total DNA were extracted using TRIzol reagent (Thermo Fisher Scientific). Total RNA was reverse transcribed into cDNA using the SuperScript III First-Strand Synthesis System (Thermo Fisher Scientific). 5′ and 3′ end sequences of genes encoding for *P. terribilis* and *D. tinctorius* Na_V_1.4 were determined by DNA gel extraction and sequencing after rapid amplification of cDNA ends using the SMARTer RACE 5′/3′ Kit (Takara Bio) and internal primers designed from *P. terribilis* and *D. tinctorius* Na_V_1.4 S6 segment sequences (Tarvin et al., 2016). From these 5′ end and 3′ end sequences, new primers were designed from both 5′ and 3′ untranslated regions of each gene and were used to amplify full-length *P. terribilis* and *D. tinctorius* Na_V_1.4 genes by PCR using Phusion HF (New England Biolabs). PCR products were gel extracted and sequenced to determine the full-length *P. terribilis* and *D. tinctorius* Na_V_1.4 gene sequences. Direct cloning of the full-length PCR products of *P. terribilis* and *D. tinctorius* Na_V_1.4 channel genes into pCDNA3.1 proved problematic, resulting in unstable constructs prone to deletion. The codon-optimized genes were synthesized for expression in human embryonic kidney cells (HEK293; GenScript) but were also found to be prone to recombination upon insertion into pCDNA3.1. Finally, the gene sequences were redesigned to differ as much as possible from the original genes, synthesized (GenScript), and cloned into pCDNA3.1. This strategy yielded stable constructs.

### Subcloning and site-directed mutagenesis

For electrophysiology experiments, *Homo sapiens* (*Hs*) Na_V_1.4 (GenBank accession no. NM_000334.4), human Na_V_β1 (GenBank accession no. NM_001037.5), *Pum* Na_V_1.4, *Pum* Na_V_β2, *Pum* Na_V_1.5, *P. terribilis* (*Pt*) Na_V_1.4, and *D. tinctorius* (*Dt*) Na_V_1.4 were subcloned into pCDNA3.1 and *Rattus norvegicus* (*Rn*) Na_V_1.4 (GenBank accession no. Y17153.1) was subcloned into pZem228. All mutants were made using the QuikChange Site-Directed Mutagenesis Kit (Agilent) and validated by complete sequencing of the genes encoding for the proteins of interest.

### Patch-clamp electrophysiology

HEK293 cells and Chinese hamster ovary (CHO) cells were grown at 37°C and 5% CO_2_ in culture medium (Dulbecco’s modified Eagle’s medium for HEK293 cells or Kaighn’s modified Ham’s F-12 medium for CHO cells) supplemented with 10% FBS, 10% L-glutamine, and antibiotics (100 IU ml^−1^ penicillin and 100 mg ml^−1^ streptomycin). HEK293 cells were transfected (in 35-mm-diameter wells) using Lipofectamine 2000 (Invitrogen) and plated onto coverslips coated with Matrigel (BD Biosciences). Human and *Pitohui* Na_V_s were coexpressed with enhanced GFP (EGFP) and human Na_V_β1 or *Pitohui* Na_V_β2. Poison frog Na_V_s were coexpressed with EGFP. Transfected cells were identified visually by EGFP expression. A total of 2 µg plasmid DNA (20% Na_V_α, 40% Na_V_β, 40% EGFP) was transfected, except for the poison frog Na_V_s, for which a total of 3 µg plasmid DNA (70% Na_V_α, 15% EGFP, 15% SV40 T antigen) was used to increase current amplitude. For mock transfections, the Na_V_α-encoding plasmid was replaced by an empty pcDNA3.1+ plasmid. Experiments designed for studying *Rn* Na_V_1.4 constructs were conducted using CHO cells cultured as described previously (Andresen and Du Bois, 2009). Briefly, cells were grown in Dulbecco’s modified Eagle’s medium (Gibco) supplemented with 10% cosmic calf serum (HyClone Laboratories) and 100 U/ml penicillin-streptomycin (Gibco). Cells were kept in a 5% CO_2_ and 96% relative humidity incubator. CHO cells were transfected in a 10-cm plate using the calcium phosphate precipitation method and EGFP expression as a marker of transfection.

Na^+^ currents were recorded by whole-cell patch clamping (Hamill et al., 1981) at room temperature (23 ± 2°C) 48–72 h after transfection. Data collection was performed using an Axopatch 200B amplifier (Molecular Devices) and pCLAMP 9 software (Molecular Devices).

Pipettes were pulled from borosilicate glass capillaries (TW150F-3; World Precision Instruments) and polished with a microforge (MF-900; Narishige) to obtain 1.2–3.5-MΩ resistances. Whole-cell access resistance was 3–8 MΩ, pipette capacitance was fully compensated, and 65–80% of the voltage error due to the series resistance was compensated. For experiments with HEK293 cells, pipette solution contained the following in mM: 120 Cs methane sulfonate, 8 NaCl, 10 EGTA, 2 Mg-ATP, and 20 HEPES (pH 7.4 with CsOH). Bath solution contained the following in mM: 155 NaCl, 1 CaCl_2_, 1 MgCl_2_, 5 KCl, 10 HEPES, and 10 glucose (pH 7.4 with NaOH). For experiments with CHO cells, pipette solution contained the following in mM: 125 CsCl, 40 NaF, 1 EDTA, and 20 HEPES (pH 7.4 with CsOH). Bath solution contained the following in mM: 160 NaCl, 2 CaCl_2_, and 20 HEPES (pH 7.4 with NaOH).

For experiments with human, bird, and frog channels, voltage-dependent activation was assessed by stimulating the cells with a multistep depolarization protocol from −90 to +50 mV using 5-mV increments, a −100-mV holding potential, and a sweep-to-sweep interval duration of 2 s. Voltage-dependent steady-state inactivation was assessed by stimulating the cells with a 500-ms prepulse depolarization from −110 to 0 mV in 5-mV steps, followed by a 20-ms step to 0 mV, and repolarization to the holding potential, −100 mV; sweep-to-sweep interval duration was 4 s. To examine BTX effects, cells were stimulated upon BTX exposure by applying at least 120 step pulses from −120 to 0 mV at 2-Hz frequency in order to facilitate BTX access into the channel pore because BTX is known to preferentially interact with the open state of Na_V_s (Tanguy and Yeh, 1991). For experiments with rat Na_V_1.4, voltage-dependent activation was assessed by stimulating the cells with a multistep depolarization protocol from −120 to +50 mV using 5-mV increments and a −120-mV holding potential. Voltage-dependent steady-state inactivation was assessed by stimulating the cells with a 150-ms prepulse depolarization from −140 to 0 mV in 5-mV steps, followed by a 50-ms step to 0 mV, and repolarization to the holding potential, −120 mV. Equilibration of BTX was accomplished with persistent activation of channels by applying a 24-ms step depolarization from −120 to 0 mV at a frequency of 2 Hz over the course of 8 min. Leak currents were subtracted using a P/4 protocol during data acquisition. Data analysis was performed using Clampfit 10.6 (Axon Instruments).

Activation curves were obtained by fitting the data with the following single or double Boltzmann equations: I = I_max_/{1 + exp[(V_1/2_ − V_m_)/k]} or I = {I_max1_/[1+exp({V_1/2,__1_ − V_m_}/k_1_)]} + {I_max__2_/[1 + exp({V_1/2,__2_ − V_m_}/k_2_)]}, where I_max_ is the maximal current after normalization to the driving force, V_1/2_ is the half-activation potential, V_m_ is the membrane potential, and k is the slope factor. Inactivation curves were obtained by fitting the data with the following single Boltzmann equation: I = I_max_/{1 + exp[(V_m_ − V_1/2_)/k]}, where I_max_ is the absolute value of the maximal current at the test pulse, V_1/2_ is the half-inactivation potential, V_m_ is the membrane potential, and k is the slope factor. Current density was determined as the ratio between current amplitude and the membrane capacitance. In cells transfected with Na_V_ constructs, green cells having no apparent Na_V_ currents were extremely rare and therefore were not included in current density assessment. All cells with a whole-cell access resistance >8 MΩ or a leak current more negative than −200 pA were excluded.

### Two-electrode voltage-clamp electrophysiology

Two-electrode voltage-clamp recordings were performed on defolliculated stages V–VI *Xenopus laevis* oocytes harvested (under UCSF IACUC protocol AN178461) 1–2 d after microinjection with mRNA. Linearized *Pitohui* (*Pum*), *Pt*, or *Dt* Na_V_1.4 cDNA was translated into capped mRNA using the mMESSAGE mMACHINE T7 Transcription Kit (Invitrogen). *Xenopus* oocytes were injected with 0.5–2 ng, 3–6 ng, or 10–30 ng of *Pum* Na_V_1.4, *Pt* Na_V_1.4, or *Dt* Na_V_1.4 mRNA, respectively. Two-electrode voltage-clamp experiments were performed 1–2 d after injection. Data were acquired using a GeneClamp 500B amplifier (MDS Analytical Technologies) controlled by pClamp software (Molecular Devices) and digitized at 1 kHz using a Digidata 1332A digitizer (MDS Analytical Technologies).

Oocytes were impaled with borosilicate recording microelectrodes (0.3–3.0-MΩ resistance) backfilled with 3 M KCl. Sodium currents were recorded using a bath solution (RS) containing the following in mM: 96 NaCl, 1 CaCl_2_, 1 MgCl_2_, 2 KCl, and 5 HEPES (pH 7.5 with NaOH) supplemented with antibiotics (50 µg ml^−1^ gentamicin, 100 IU ml^−1^ penicillin, and 100 µg ml^−1^ streptomycin) and 2.5 mM sodium pyruvate.

For studying the competition between tricaine and BTX, 0.5 mM tricaine was applied by continuous perfusion in the bath solution to assess channel block. BTX was applied from the intracellular side of the membrane by injecting oocytes with 50 nl of 2 mM BTX. After BTX injection, oocytes were stimulated by applying 1,000 step pulses of 60 ms each, from −120 to 0 mV at 2 Hz frequency, in order to facilitate BTX access into the channel pore.

To determine STX and TTX dose–response curves, solutions containing test concentrations of each toxin were applied in series by perfusion to oocytes expressing *Pum* Na_V_1.4, *Pt* Na_V_1.4, or *Dt* Na_V_1.4. IC_50_ values were calculated from the ratio of peak currents in the presence and absence of toxin, based on the following equation: $\frac{I_{x}}{I_{0}}=\frac{\left(I_{max}-I_{min}\right)}{\left(1+\frac{x}{IC_{50}}\right)},$ where I_x_ is the current amplitude at the toxin concentration *x*, I_0_ is the current amplitude in absence of toxin, and I_max_ and I_min_ are the maximum and minimum peak current amplitudes, respectively, and IC_50_ is the half-maximal inhibitory concentration.

To determine the effects of Sxph on *Pt* Na_V_1.4 STX responses, *Rana catesbeiana* Sxph was expressed from a pFastBac1 vector (Invitrogen) in Sf9 cells and purified as described previously (Yen et al., 2019). Sxph concentration was determined by measuring A_280nm_ using an extinction coefficient of 96,365 M^−1^ cm^−1^ calculated using the ExPASY server (https://web.expasy.org/protparam/). For experiments in which Sxph:STX were premixed before being applied to *Xenopus* oocytes expressing *Pt* Na_V_, varied Sxph:STX ratios were made by adding purified Sxph from a 100 µM Sxph stock solution (150 mM NaCl and 10 mM HEPES, pH 7.4) to 100 nM STX in (RS) at least 10 min before perfusion. In the order of addition experiments, following recording channel behavior in the absence of the toxins, toxin concentrations to achieve ∼90% block, 100 nM STX or 300 nM TTX, in RS was applied to the channels before Sxph to the desired concentration was then added directly to a 1-ml recording chamber containing the toxin. For all [Sxph]:[STX] ratios, the concentration of the stock Sxph solution added to the chamber was adjusted so that the volume of the added Sxph solution was <1% of the total volume of the recording chamber.

All toxin effects were assessed with 60-ms depolarization steps from −120 to 0 mV with a holding potential of −120 mV and a sweep-to-sweep duration of 10 s.

Recordings were conducted at room temperature (23 ± 2°C). Leak currents were subtracted using a P/4 protocol during data acquisition. Data analysis was performed using Clampfit 10.6 (Axon Instruments) and a custom software developed in the Igor environment (Wavemetrics).

### Toxin challenge experiments

Frogs for the toxin challenge experiments were obtained from the following sources: *Polypedates leucomystax*, *P. terribilis*, and *D. tinctorius* (Josh’s Frogs); *Xenopus* (Nasco); and *Mantella aurantiaca* (Indoor Ecosystems). All experiments were performed in accordance with UCSF IACUC protocol AN136799.

Frogs were held at room temperature (23 ± 2°C) and anesthetized with a 0.15% tricaine (MS-222) bath before toxin injections. Once under anesthesia, as judged by immobility and lack of response to foot pinching, frogs were weighed in order to calculate the appropriate amount of toxin to be administered at 20 times the LD_50_ based on the values calculated for mice as follows: BTX, 2 µg/kg (Albuquerque et al., 1971); STX, 10 µg/kg (Wiberg and Stephenson, 1960); and TTX, 12.5 µg/kg (Lago et al., 2015). BTX, STX, and TTX were delivered using 40 ng, 200 ng, and 250 ng of toxin, respectively, per gram of animal weight. The upper right hind leg was injected with either control, PBS, or toxin-containing solution under an SMZ645 binocular microscope (Nikon) using a 30-gauge PrecisionGlide needle (BD Biosciences). BTX, STX, or TTX dissolved in PBS was injected at the appropriate concentration to deliver 20 times the LD_50_. The total volume of injection was 100 µl in *Xenopus* and 10 µl in other frogs due to their smaller size. The choice of intramuscular injection was to avoid internal organ damage. Frogs were allowed to recover in a separate container and monitored constantly for signs of recovery, paralysis, or other adverse symptoms. For *Xenopus*, the recovery container was filled with deionized water and inclined in a way that the frogs could recover on the dry surface of the container base. Postrecovery activity was then assessed by the ability of the frogs to move from the dry to the water-containing surface because *Xenopus* are primarily aquatic animals. For all other frogs, postrecovery activity was assessed by monitoring the ability of the animals to put themselves right side up from a supine position in their recovery containers. The monitoring period was up to 24 h after injection; and three animals were tested for each condition.

### Online supplemental material

**Figure S1.**
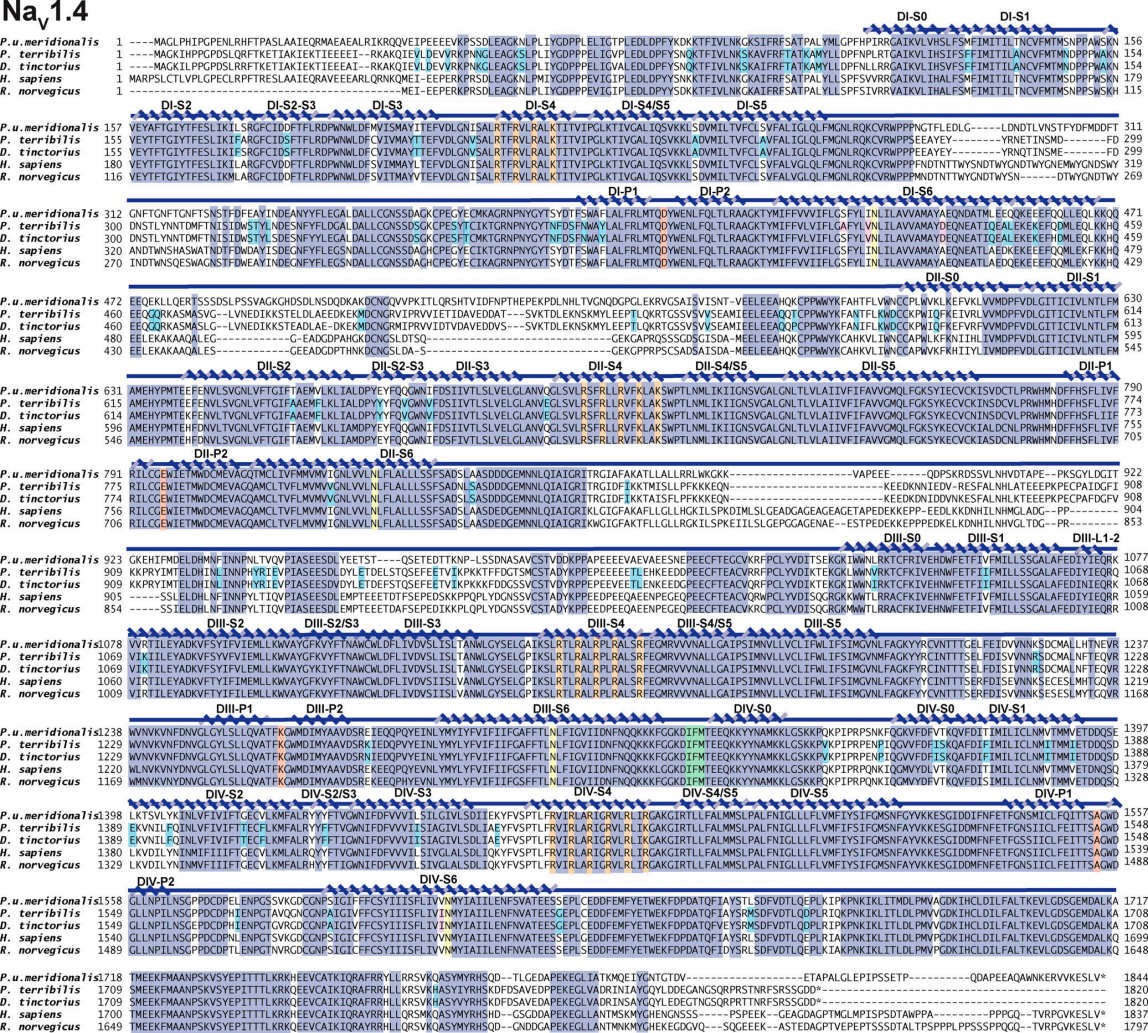
***Pitohui* and poison frog Na_V_1.4 sequences.** Sequence alignment of *Pum* Na_V_1.4, *Pt* Na_V_1.4, *Dt* Na_V_1.4, *Hs* Na_V_1.4 (RefSeq accession no. NP_000325.4), and *Rn* Na_V_1.4 (RefSeq accession no. NP_037310.1). Key Na_V_ features are highlighted as follows: selectivity filter DEKA (red), IFM peptide (green), conserved S6 Asn (yellow), S4 voltage sensor arginines (orange), poison frog variants (cyan), and sites highlighted by Tarvin et al. (2016; magenta) are indicated. Conserved residues are highlighted in dark blue. Secondary structure elements were labeled using boundaries from Yan et al. (2017).

**Figure S2.**
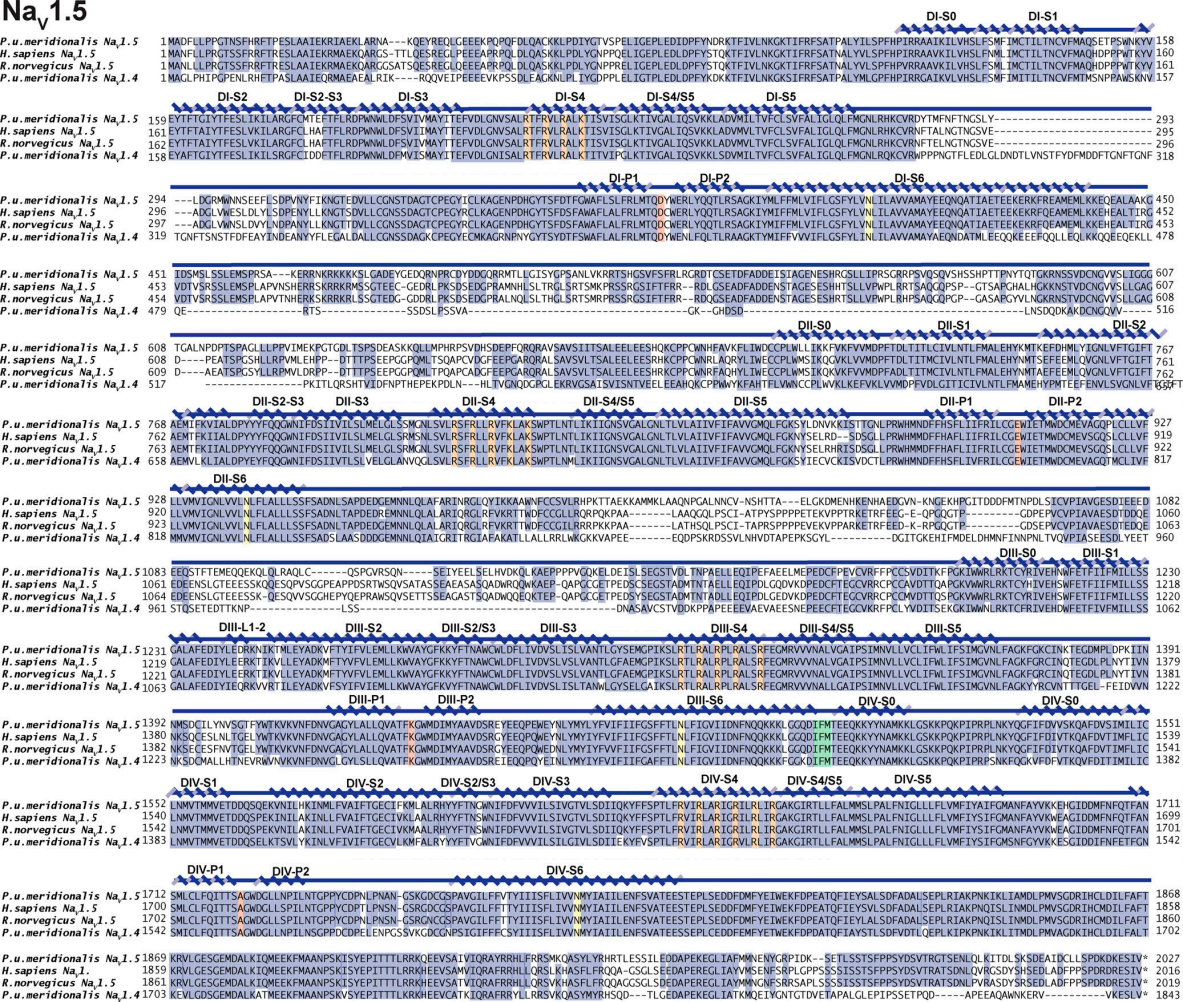
***Pitohui* Na_V_1.5 sequence.** Sequence alignment of *Pum* Na_V_1.5, *Hs* Na_V_1.5 (RefSeq accession no. NP_932173.1), *Rn* Na_V_1.5 (RefSeq accession no. NP_037257.1), and *Pum* Na_V_1.4. Key Na_V_ features are highlighted as follows: selectivity filter DEKA (red), IFM peptide (green), conserved S6 Asn (yellow), and S4 voltage sensor arginines (orange). Conserved residues are highlighted in dark blue. Secondary structure elements were labeled using boundaries from Yan et al. (2017).

**Figure S3.**
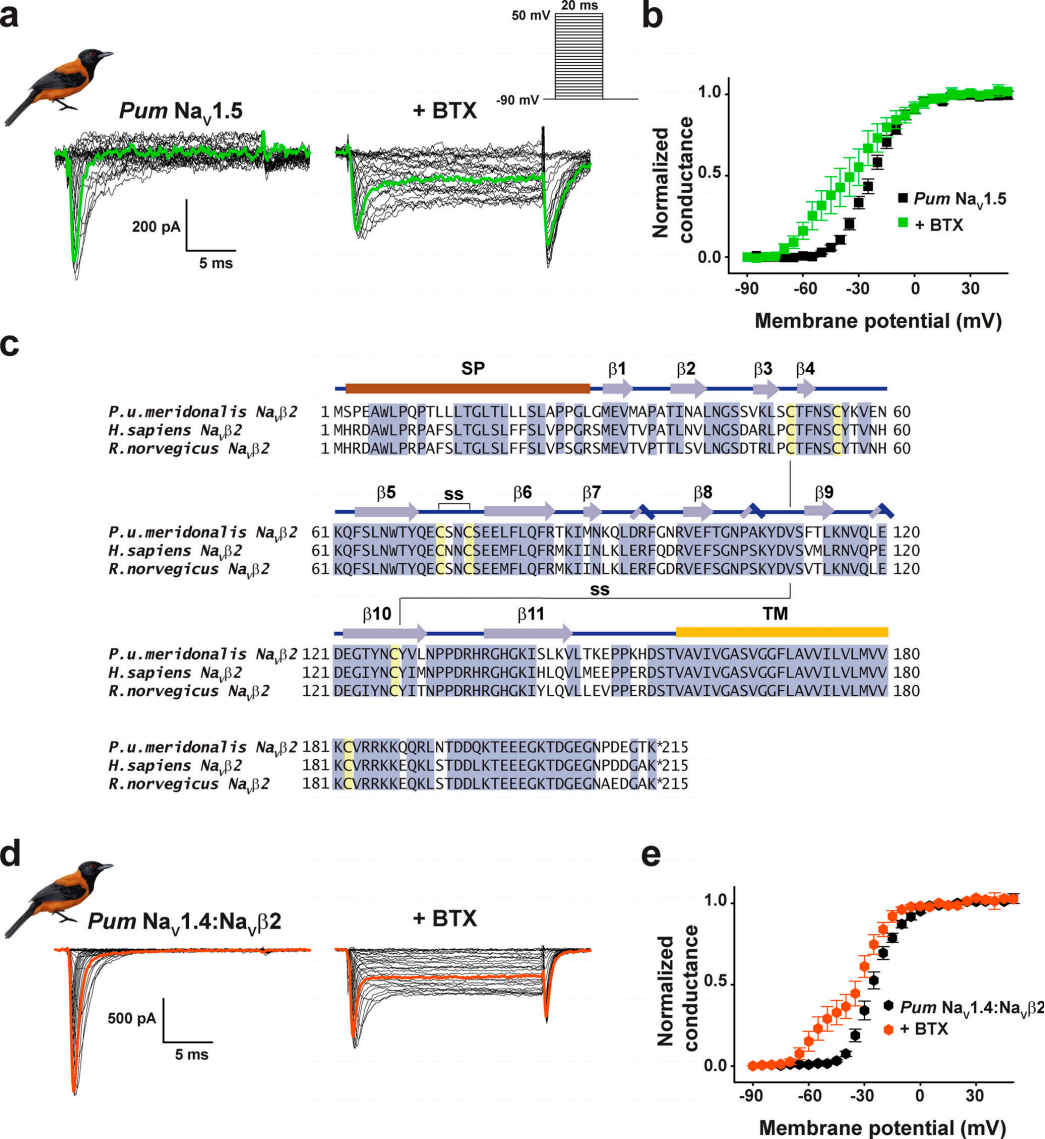
***Pitohui* Na_V_1.5 and Na_V_1.4:Na_V_β2 complexes are BTX sensitive. (a)** Exemplar current recordings for *Pum* Na_V_1.5 expressed in HEK293 cells in the absence (left) or presence (right) of 10 µM BTX. Trace at 0 mV is highlighted in each panel. Currents were evoked with the shown multistep depolarization protocol (inset). **(b)** G-V relationships in the absence (black squares) or presence (green squares) of 10 µM BTX. **(c)** Sequence alignment of Na_V_β2 from *Pum, Hs* (RefSeq accession no. NP_004579.1), and *Rn* (RefSeq accession no. NP_037009.1). Signal peptide (SP), secondary structural elements from Das et al. (2016), conserved disulfide bond (ss), and transmembrane domain (TM) are indicated. **(d)** Exemplar current recordings for *Pum* Na_V_1.4:Na_V_β2 expressed in HEK293 cells in the absence (left) or presence (right) of 10 µM BTX. Trace at 0 mV is highlighted in each panel. Currents were evoked with the shown multistep depolarization protocol (inset in a). **(e)** G-V relationships in the absence (black hexagons) or presence (red hexagons) of 10 µM BTX.

**Figure S4.**
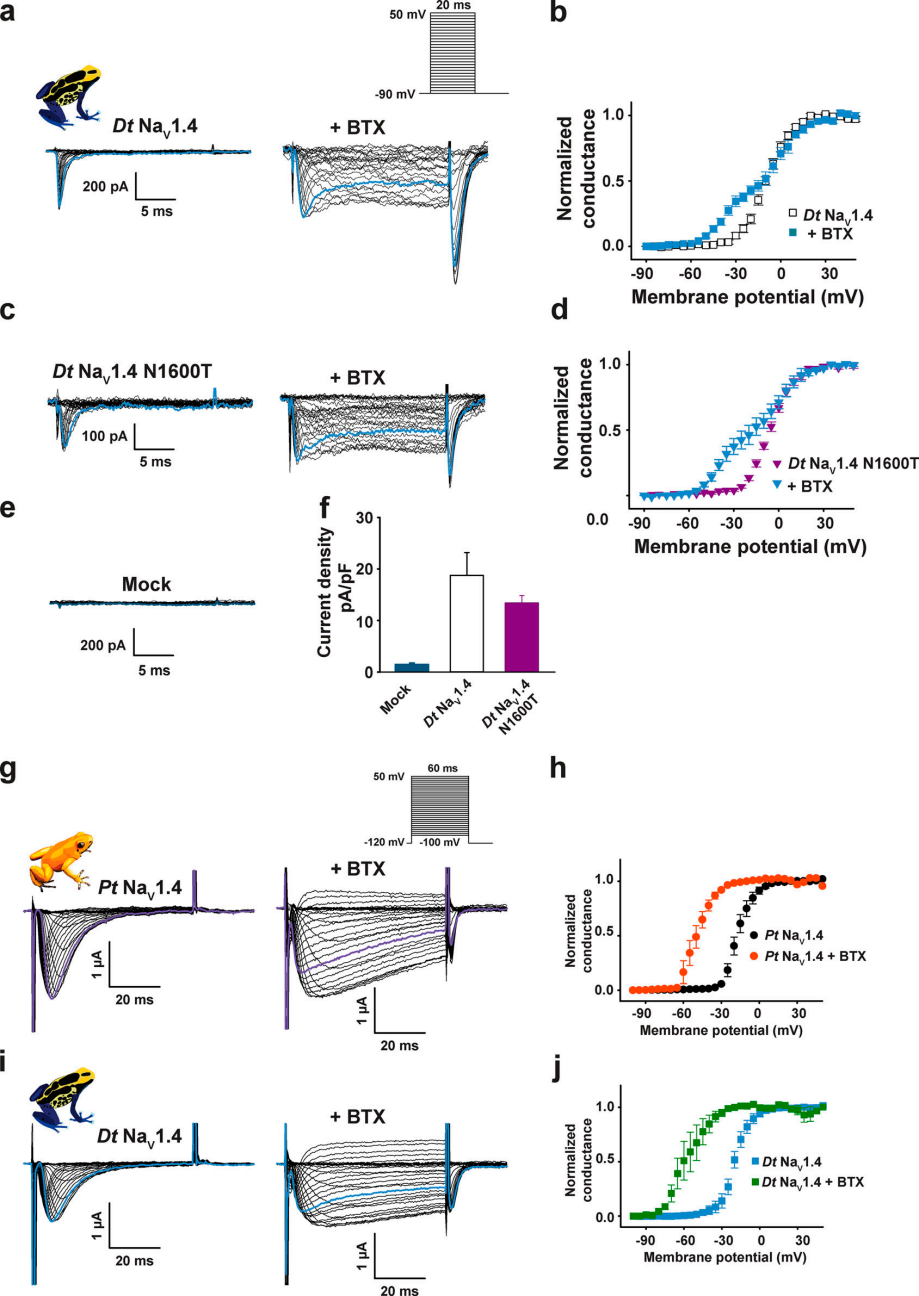
**Poison frog Na_V_1.4s expressed in CHO cells and *Xenopus* oocytes are BTX sensitive.(a, c, and g)** Exemplar current recordings for *Dt* Na_V_1.4 (a) and *Dt* Na_V_1.4 N1600T (c) expressed in CHO cells in the absence (left) or presence (right) of 10 µM BTX. Currents were evoked with the shown multistep depolarization protocol (inset in a). **(e)** Currents from mock-transfected cells using the same protocol as for a and c. Trace at 0 mV is highlighted in each panel. **(b, and d)** G-V relationships in the presence or absence of 10 µM BTX; *Dt* Na_V_1.4 (open squares), +BTX (light blue squares; b); and *Dt* Na_V_1.4 N1600T (purple inverted triangles; d), +BTX (light blue inverted triangles; j). **(f)** Current densities for mock-transfected cells (blue), *Dt* Na_V_1.4 (white), and *Dt* Na_V_1.4 N1600T (purple). **(g and i)** Exemplar two-electrode voltage clamp (TEVC) current recordings for *Pt* Na_V_1.4 (g) and *Dt* Na_V_1.4 (i) expressed in *Xenopus* oocytes in the absence (left) or presence (right) of 10 µM BTX. Trace at 0 mV is highlighted in each panel. Currents were evoked with the shown multistep depolarization protocol (inset in g). **(h and j)** G-V relationships in the presence or absence of 10 µM BTX, *Pt* Na_V_1.4 (black circles), +BTX (orange circles; for h); and *Dt* Na_V_1.4 (light blue squares), +BTX (green squares; j).

**Figure S5.**
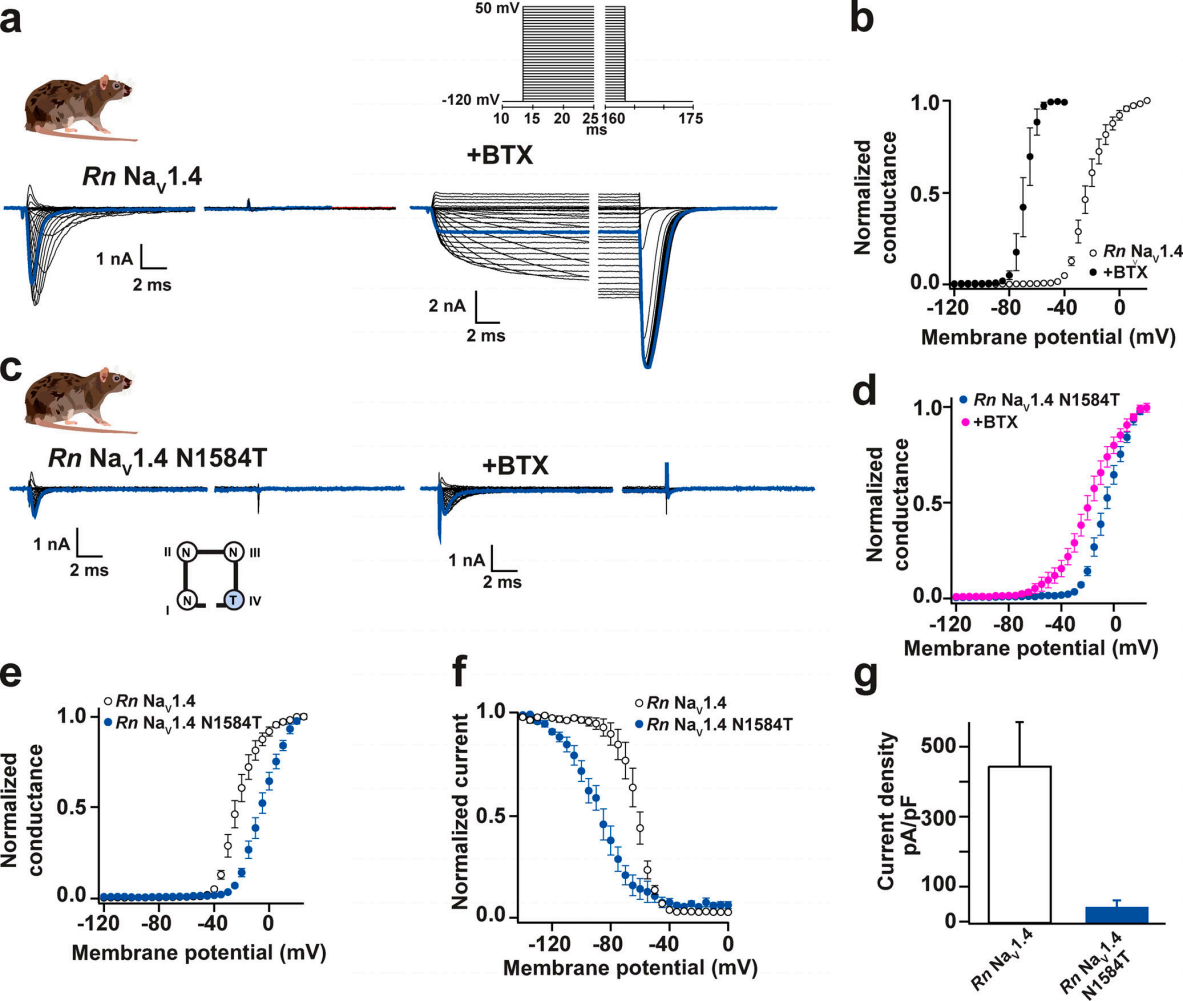
**Functional costs of DIV-S6 Asn mutation in *Rn* Na_V_1.4.(a and c)** Exemplar current recordings for *Rn* Na_V_1.4 (a) and *Rn* Na_V_1.4 N1584T (c) expressed in CHO cells in the absence (left) or presence (right) of 10 µM BTX. Trace at 0 mV is highlighted in each panel. Currents were evoked with the shown multistep depolarization protocol (inset in a). **(b and d)** G-V relationships in the presence or absence of 10 µM BTX, *Rn* Na_V_1.4 (open circles), +BTX (black circles; b); and *Rn* Na_V_1.4 N1584T (blue circles), +BTX (magenta circles; d). **(e)** G-V relationships. **(f)** Steady-state inactivation voltage dependencies for *Rn* Na_V_1.4 (open circles) and *Rn* Na_V_1.4 N1584T (blue circles). **(g)** Current densities for *Rn* Na_V_1.4 (white) and *Rn* Na_V_1.4 N1584T (blue).

**Figure S6.**
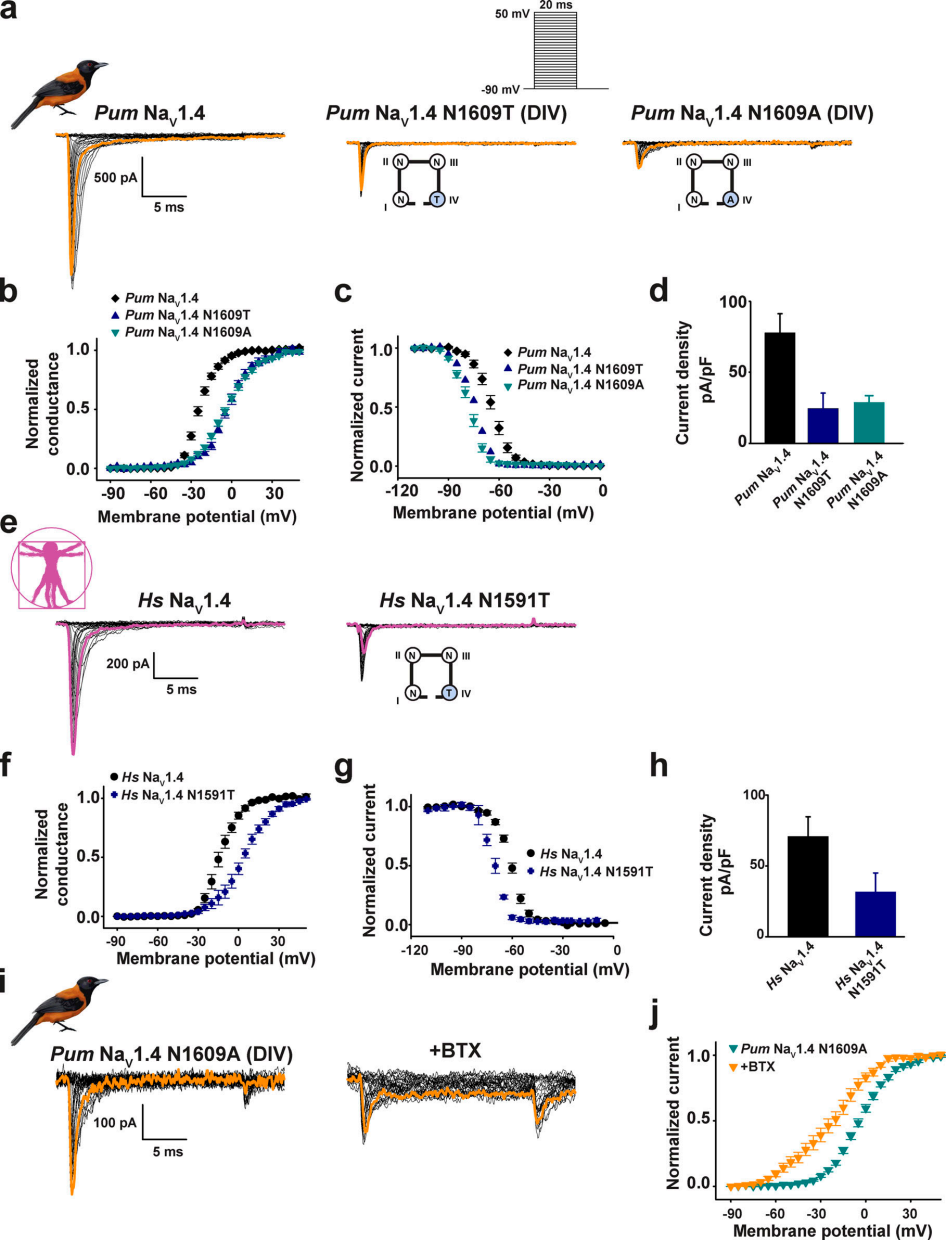
**Functional cost of DIV-S6 Asn mutation in *Pum* Na_V_1.4 and *Hs* Na_V_1.4.(a)** Exemplar current recordings for *Pum* Na_V_1.4 (left), *Pum* Na_V_1.4 N1609T (middle), and *Pum* Na_V_1.4 N1609A (right) expressed in HEK293 cells. Trace at 0 mV is highlighted, and currents were evoked with the shown multistep depolarization protocol (inset). Cartoon shows a diagram of the identities of the S6 Asn for the Asn mutants. **(b–d)** G-V relationships (b). Steady-state inactivation voltage dependencies (c), and current densities (d) for *Pum* Na_V_1.4 (black diamonds), *Pum* Na_V_1.4 N1609T (blue triangles), and *Pum* Na_V_1.4 N1609A (teal inverted triangles). **(e)** Exemplar current recordings for *Hs* Na_V_1.4 (left), *Hs* Na_V_1.4 N1591T (right), expressed in HEK293 cells. Trace at 0 mV is highlighted. Currents were evoked with the shown multistep depolarization protocol from a. **(f–h)** G-V relationships (f), steady-state inactivation voltage dependencies (g), and current densities (h) for *Hs* Na_V_1.4 (black circles) and *Hs* Na_V_1.4 N1591T (blue diamonds). **(i)** Exemplar current recordings for *Pum* Na_V_1.4 N1609A (left) and in the presence of 10 µM BTX (right). **(j)** G-V relationships for *Pum* Na_V_1.4 N1609A (green inverted triangles) and in the presence of 10 µM BTX (orange inverted triangles).

**Figure S7.**
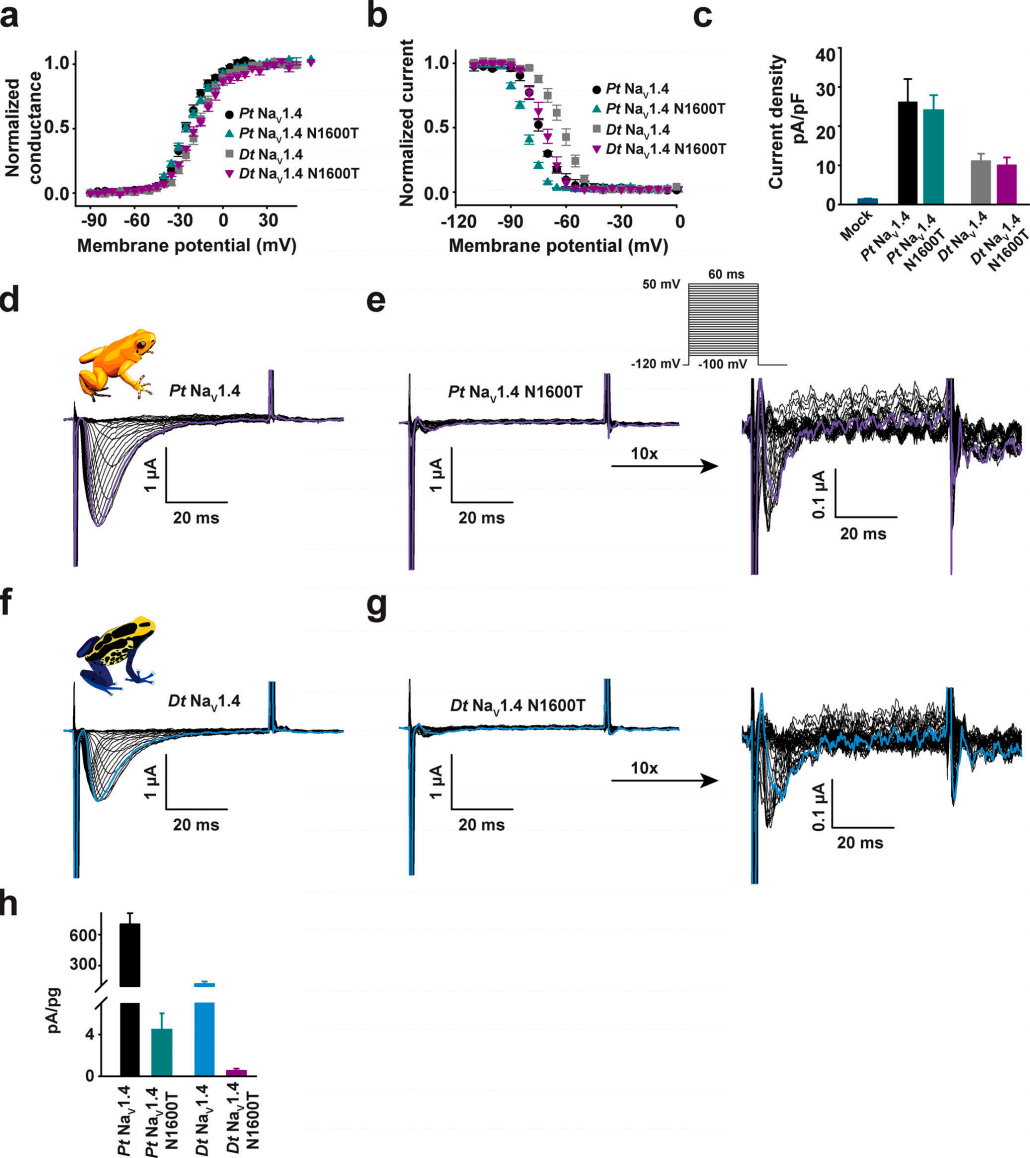
**Functional cost of DIV-S6 N→T mutation in poison frog Na_V_1.4s. (a–c)** G-V relationships (a), steady-state inactivation voltage dependences (b), and current densities (c) for mock-transfected cells and *Pt* Na_V_1.4 (black circles), *Pt* Na_V_1.4 N1600T (cyan triangles), *Dt* Na_V_1.4 (grey squares), and *Dt* Na_V_1.4 N1600T (magenta downward triangles) expressed in HEK293 cells. **(d–g)** Exemplar current recordings for *Pt* Na_V_1.4 (d), *Pt* Na_V_1.4 N1600T (e), *Dt* Na_V_1.4 (f), and *Dt* Na_V_1.4 N1600T (g) expressed in *Xenopus* oocytes. 10× magnifications of *Pt* Na_V_1.4 N1600T and *Dt* Na_V_1.4 N1600T traces are shown in e and g, right panels. Trace at 0 mV is highlighted in each panel. Currents were evoked with the shown multistep depolarization protocol (inset in e). **(h)** Current amplitudes normalized to the amount of injected RNA for the indicated poison frog constructs.

**Figure S8.**
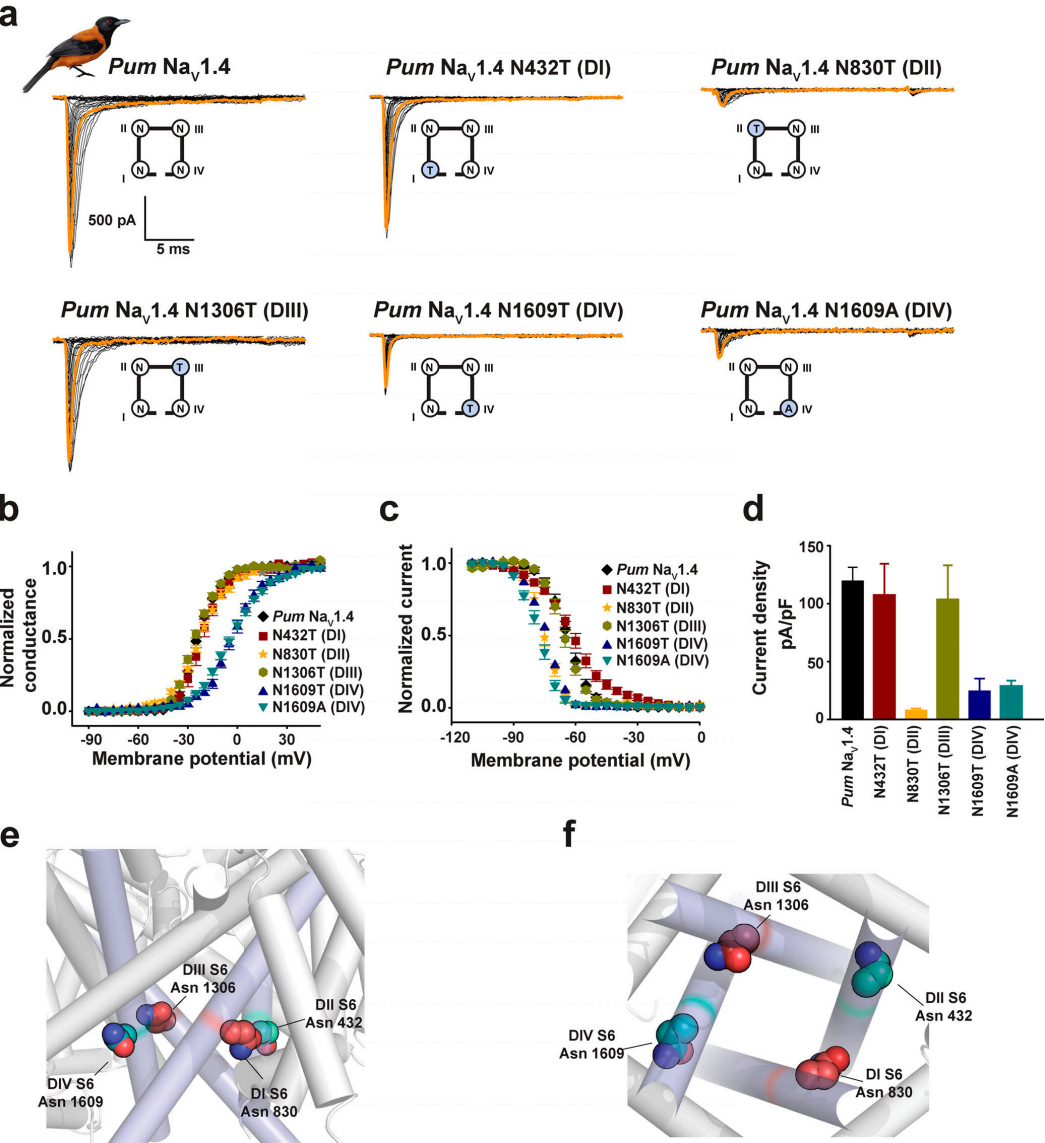
**Functional studies of S6 Asn mutants support asymmetric properties of the channel pore. (a–d)** Exemplar current recordings (a), G-V relationships (b), steady-state inactivation voltage dependences (c), and current densities (d) for *Pum* Na_V_1.4 (black diamonds), *Pum* Na_V_1.4 N432T (dark red squares), *Pum* Na_V_1.4 N830T (orange stars), *Pum* Na_V_1.4 N1306T (green hexagons), *Pum* Na_V_1.4 N1609T (dark blue triangles), and *Pum* Na_V_1.4 N1609A (cyan downward triangles) expressed in HEK293 cells. Trace at 0 mV is highlighted in each panel. Cartoon shows a diagram of the identities of the S6 Asn for each construct. **(e and f)** Side (e) and bottom (f) views of the locations the S6 conserved asparagines. Residues are mapped on the structure of human Na_V_1.4 (Protein Data Bank accession no. 6ADF; (Pan et al., 2018) and are labeled using the *Pum* Na_V_1.4 numbering.

Fig. S1 shows *Pitohui* and poison frog Na_V_1.4 sequences. Fig. S2 shows the *Pitohui* Na_V_1.5 sequence. Fig. S3 shows that *Pitohui* Na_V_1.5 and Na_V_1.4:Na_V_β2 complexes are BTX sensitive. Fig. S4 shows that poison frog Na_V_1.4s expressed in CHO cells and *Xenopus* oocytes are BTX sensitive. Fig. S5 shows the functional costs of DIV-S6 Asn mutation in *Rn* Na_V_1.4. Fig. S6 shows the functional cost of DIV-S6 Asn mutation in *Pum* Na_V_1.4 and *Hs* Na_V_1.4. Fig. S7 shows the functional cost of DIV-S6 N→T mutation in poison frog Na_V_1.4s. Fig. S8 shows functional studies of S6 Asn mutants that support asymmetric properties of the channel pore. Table S1 lists Na_V_ inactivation parameters. Table S2 lists human → poison frog Na_V_1.4 amino acid variants. Table S3 shows the recovery time from anesthesia. Data S1 provides gene assembly scripts.

## Results

### *Pitohui* poison birds and poison frogs have BTX-sensitive Na_V_s

*Pitohui* is one of only a few bird genera known to carry BTX (Dumbacher et al., 1992; Dumbacher et al., 2000; Menon and Dumbacher, 2014) and has BTX levels in its skeletal and cardiac muscles that should alter Na_V_ function (∼5 and ∼20 µM, respectively; Dumbacher et al., 2009; MacKenzie et al., 2021 *Preprint*). To investigate possible mechanisms of BTX resistance, we used a *Pitohui* genomic DNA library to identify and assemble genes for *Pum* skeletal muscle Na_V_1.4 (Fig. S1) and cardiac *Pum* Na_V_1.5 (Fig. S2). Primary sequence alignment showed extensive similarities between *Pum* Na_V_1.4, *Pum* Na_V_1.5, and other vertebrate homologues (∼73% amino acid identity; Figs. S1 and S2), including hallmark Na_V_ features such as a selectivity filter aspartate-glutamate-lysine-alanine (DEKA) motif, canonical RXXR repeats in S4 in all four voltage sensor domains, and the isoleucine-​phenylalanine-methionine (IFM) motif responsible for fast inactivation (Catterall et al., 2020).

**Figure 1.**
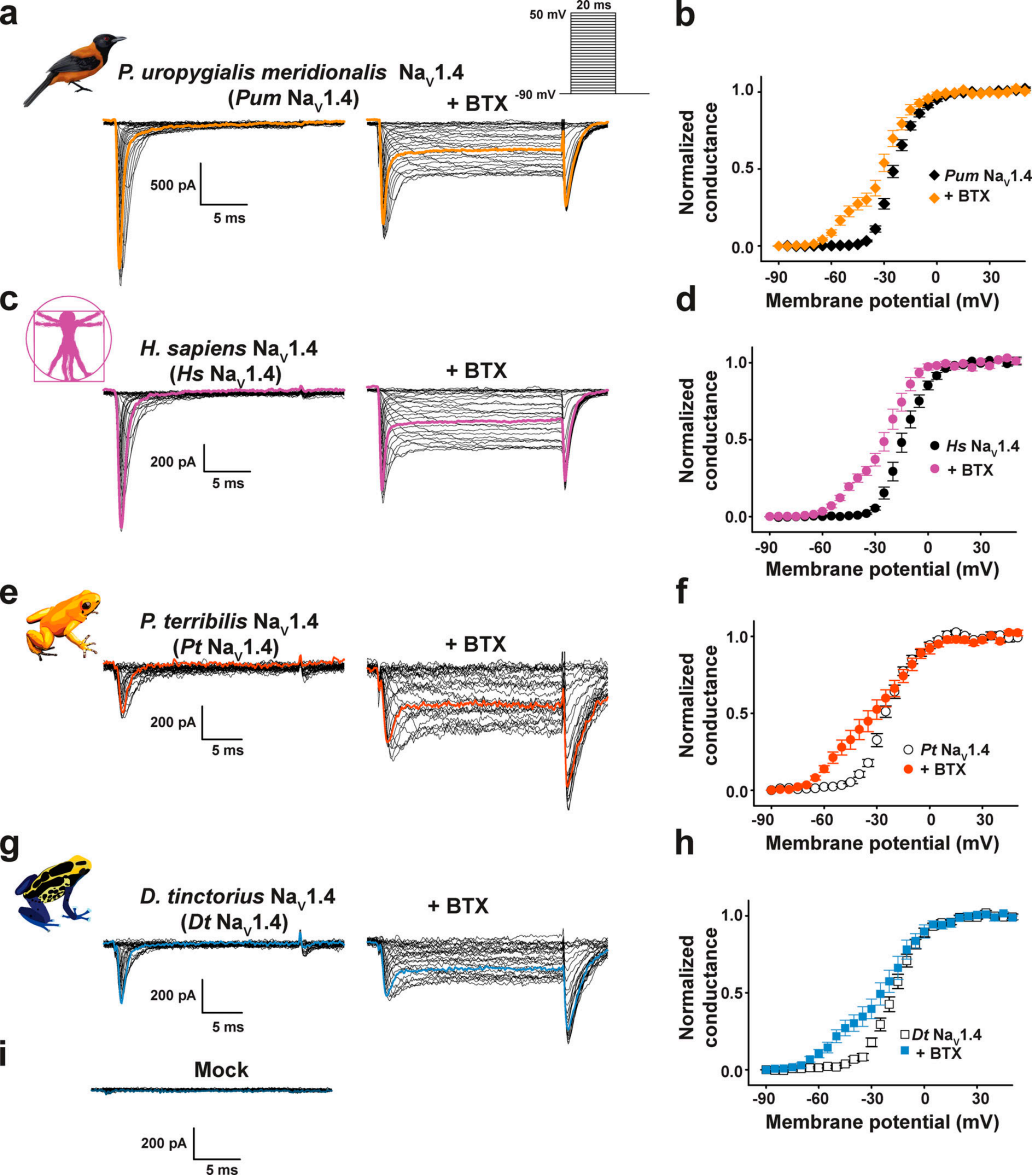
***Pitohui* and poison frog Na_V_1.4 channels are BTX sensitive.(a, c, e, and g)** Exemplar current recordings for *Pum* Na_V_1.4 (a), *Hs* Na_V_1.4 (c), *Pt* Na_V_1.4 (e), and *Dt* Na_V_1.4 (g) expressed in HEK293 cells in the absence (left) or presence (right) of 10 µM BTX. Trace at 0 mV is highlighted in each panel. Currents were evoked with the shown multistep depolarization protocol (inset in a). **(b, d, f, and h)** G-V relationships in the presence or absence of 10 µM BTX for *Pum* Na_V_1.4 (black diamonds), +BTX (orange diamonds; b), *Hs* Na_V_1.4 (black circles), +BTX (purple circles; d), *Pt* Na_V_1.4 (white circles), +BTX (dark orange circles; f), and *Dt* Na_V_1.4 (white squares), +BTX (blue squares; h). **(i)** Exemplar current recordings from mock-transfected HEK293 cells using the protocol from a.

Whole-cell patch-clamp electrophysiology of *Pum* Na_V_1.4 and *Pum* Na_V_1.5 transfected into HEK293 cells demonstrated that both have fast voltage-dependent activation followed by a fast and complete voltage-dependent inactivation typical of Na_V_s (Fig. 1, a and b; Fig. S3, a and b; Table 1; and Table S1), similar to *Hs* Na_V_1.4 recorded under identical conditions (Fig. 1, c and d; Table 1; and Table S1). Because Na_V_s can harbor resistance mutations to other small-molecule toxins (Arbuckle et al., 2017; Almabruk et al., 2018; Geffeney et al., 2002; Bricelj et al., 2005), we anticipated that the *Pitohui* Na_V_s might be BTX resistant. Surprisingly, application of 10 µM BTX, a concentration comparable to that found in *Pitohui* muscle (Dumbacher et al., 2009), drastically altered the function of both *Pum* Na_V_s, yielding typical BTX-induced functional consequences: a hyperpolarized shift in the voltage dependency of activation (ΔV_1/2 BTX_ = −33.6 ± 1.2 and −37.4 ± 1.8 mV for *Pum* Na_V_1.4 and *Pum* Na_V_1.5, respectively), reduced inactivation, and enhanced tail currents (Khodorov, 1985; Logan et al., 2016; Fig. 1, a and b; Fig. S3, a and b; and Table 1). The BTX-induced activation curve follows a double Boltzmann function in which the first and second components arise from BTX-bound and unmodified channels, respectively (Du et al., 2011). Notably, the BTX-induced changes were equivalent to those elicited by BTX application to *Hs* Na_V_1.4 (ΔV_1/2 BTX_ = −35.9 ± 1.8 mV; Fig. 1 d and Table 1). Na_V_s are often coexpressed with auxiliary β-subunits that can alter their biophysical (Calhoun and Isom, 2014) and pharmacological (Gilchrist et al., 2014; Zhang et al., 2013) properties. To test whether this subunit could affect BTX resistance, we identified the *Pum* gene encoding for a transmembrane protein bearing the key features of Na_V_β2 (Das et al., 2016; Fig. S3 c). Cotransfection of *Pum* Na_V_β2 with *Pum* Na_V_1.4 had no impact on channel biophysical properties or on BTX responses (Fig. S3, d and e; Table 1; and Table S1). Thus, *Pum* Na_V_1.4 alone and *Pum* Na_V_1.4 in combination with *Pum* Na_V_β2 failed to show evidence of BTX-resistant channels. Together, these data demonstrate that even though *Pitohui* carry BTX in their skeletal muscles and heart (Dumbacher et al., 2009), their skeletal and cardiac Na_V_s are BTX sensitive. Thus, autoresistance cannot originate from altered BTX sensitivity in the two most likely targets exposed to lethal BTX levels.

**Table 1.** Activation parameters

Channel		Current density (pA/pF)^a^ (pA/pg)^b^	V_1/2_-I (mV)	ΔV_1/2 BTX_ (mV)	k_act_-I (mV)	V_1/2_-II (mV)	k_act_-II (mV)	*n*
***Bird***	***Pum* Na_V_1.4**	119.5 ± 11.8	−23.4 ± 1.0		5.9 ± 0.3			20
	+BTX		−57.0 ± 0.7	−33.6 ± 1.2	3.8 ± 0.6	−26.0 ± 1.7	5.9 ± 0.8	9
	***Pum* Na_V_1.4 + ** *Pum* ** Na_V_β2**	86.5 ± 12.8	−24.9 ± 1.3		6.2 ± 0.5			15
	+BTX		−59.9 ± 1.9	−35.0 ± 2.3	3.5 ± 0.5	−28.0 ± 1.4	5.7 ± 0.6	7
	***Pum* Na_V_1.5**	17.1 ± 3.5	−22.4 ± 1.6		8.6 ± 0.3			9
	+BTX		−59.8 ± 0.8	−37.4 ± 1.8	5.5 ± 1.9	−25.0 ± 2.3	11.3 ± 0.7	8
	***Pum* Na_V_1.4 N432T (DI)**	107.5 ± 26.9	−20.2 ± 1.9		6.7 ± 0.5			7
	+BTX		−51.0 ± 0.9	−30.8 ± 2.1	4.4 ± 0.4	−9.9 ± 6.1	7.4 ± 1.4	10
	***Pum* Na_V_1.4 N830T (DII)**	8.1 ± 1.5	−22.4 ± 1.0		9.2 ± 0.5			16
	+BTX		−54.0 ± 1.7	−31.6 ± 2.0	7.6 ± 1.0	−22.9 ± 1.8	5.2 ± 0.9	9
	***Pum* Na_V_1.4 N1306T (DIII)**	103.7 ± 29.4	−26.7 ± 1.0		6.4 ± 0.3			10
	+BTX		−63.6 ± 1.0	−36.9 ± 1.4	3.7 ± 0.6	−24.8 ± 1.2	8.5 ± 1.3	5
	***Pum* Na_V_1.4 N1609T (DIV)**	24.6 ± 10.8	−2.9 ± 1.1		9.6 ± 1.0			9
	+BTX		−14.3 ± 3.0	−11.4 ± 3.2	9.7 ± 2.1	N/A	N/A	5
	***Pum* Na_V_1.4 N1609A (DIV)**	28.9 ± 4.6	−3.8 ± 1.3		10.7 ± 0.4			21
	+BTX		−45.1 ± 2.2	−41.3 ± 2.6	9.1 ± 0.8	−9.6 ± 2.0	8.6 ± 1.1	14
***Human***	***Hs* Na_V_1.4**	70.9 ± 13.8	−13.3 ± 1.6		6.1 ± 0.4			13
	+BTX		−49.2 ± 0.9	−35.9 ± 1.8	5.0 ± 0.4	−19.0 ± 1.9	5.2 ± 0.5	9
	***Hs* Na_V_1.4 N1591T (DIV)**	31.7 ± 13.4	3.6 ± 2.2		10.9 ± 1.1			4
	+BTX		−10.6 ± 5.0	−14.2 ± 5.5	9.7 ± 0.6	N/A	N/A	6
***Rat***	***Rn* Na_V_1.4**	445.0 ± 125.1	−23.1 ± 0.3		7.3 ± 0.3			4
	+BTX		−68.7 ± 0.2	−45.6 ± 0.4	4.0 ± 0.1			4
	***Rn* Na_V_1.4 N1584T (DiV)**	43.2 ± 40.1	−5.1 ± 0.5		8.6 ± 0.3			6
	+BTX		−45.4 ± 2.7	−40.3 ± 2.8	9.6 ± 1.1	−17.0 ± 1.2	13.0 ± 0.9	6
***Poison frog***	***Pt* Na_V_1.4**	26.2 ± 5.9	−24.3 ± 1.4		7.6 ± 0.3			6
	Oocytes	669.7 ± 120.4	−16.2 ± 2.0		4.3 ± 0.6			11
	+BTX		−54.3 ± 1.6	−30.0 ± 2.1	6.3 ± 0.8	−20.0 ± 2.2	9.1 ± 1.5	9
	+BTX oocytes		−49.3 ± 2.5	−33.1 ± 3.2	4.8 ± 0.7			8
	***Pt* Na_V_1.4 (N1600T) (DIV)**	24.1 ± 3.9	−23.8 ± 1.2		9.0 ± 0.3			10
	+BTX		−54.6 ± 2.2	−30.8 ± 2.5	5.6 ± 0.8	−19.0 ± 6.7	11.7 ± 2.0	6
	***Dt* Na_V_1.4**	11.1 ± 1.8	−17.1 ± 1.6		7.7 ± 0.4			13
	CHO cells	18.7 ± 4.4	−9.7 ± 1.5		8.0 ± 0.4			11
	oocytes	108.1 ± 24.3	−19.6 ± 2.5		5.1 ± 0.6			10
	+BTX		−55.0 ± 1.2	−37.9 ± 2.0	5.8 ± 1.4	−17.4 ± 2.7	8.2 ± 0.8	6
	+BTX CHO cells		−39.3 ± 1.9	−29.6 ± 2.4	6.7 ± 0.5	−0.2 ± 2.1	8.5 ± 0.9	7
	+BTX oocytes		−57.4 ± 5.6	−37.8 ± 6.1	5.2 ± 0.7			5
	***Dt* Na_V_1.4 (N1600T) (DIV)**	10.0 ± 2.0	−17.2 ± 1.2		10.7 ± 0.9			4
	CHO cells	13.4 ± 1.4	−5.5 ± 1.2		8.0 ± 0.4			12
	+BTX		−55.1 ± 1.4	−37.8 ± 1.8	6.0 ± 0.5	−18.0 ± 3.1	9.8 ± 1.3	4
	+BTX CHO cells		−40.1 ± 2.4	−34.6 ± 2.7	5.9 ± 0.8	1.4 ± 3.1	7.0 ± 1.0	7

Current densities for mock-transfected HEK293 and CHO cells were 1.3 ± 0.2 and 1.3 ± 0.3 pA/pF, *n* = 10 and 14, respectively. V_1/2_-I and V_1/2_-II are the half-activation potential. k_act_I an k_act_II are the slope factors. Values having “I” only indicate those fit by a single Boltzmann equation. Values having “I” and “II” indicate in cases where the data are fit by a double Boltzmann equation.

a Values for mammalian cells.

b Values for oocytes.

**Figure 2.**
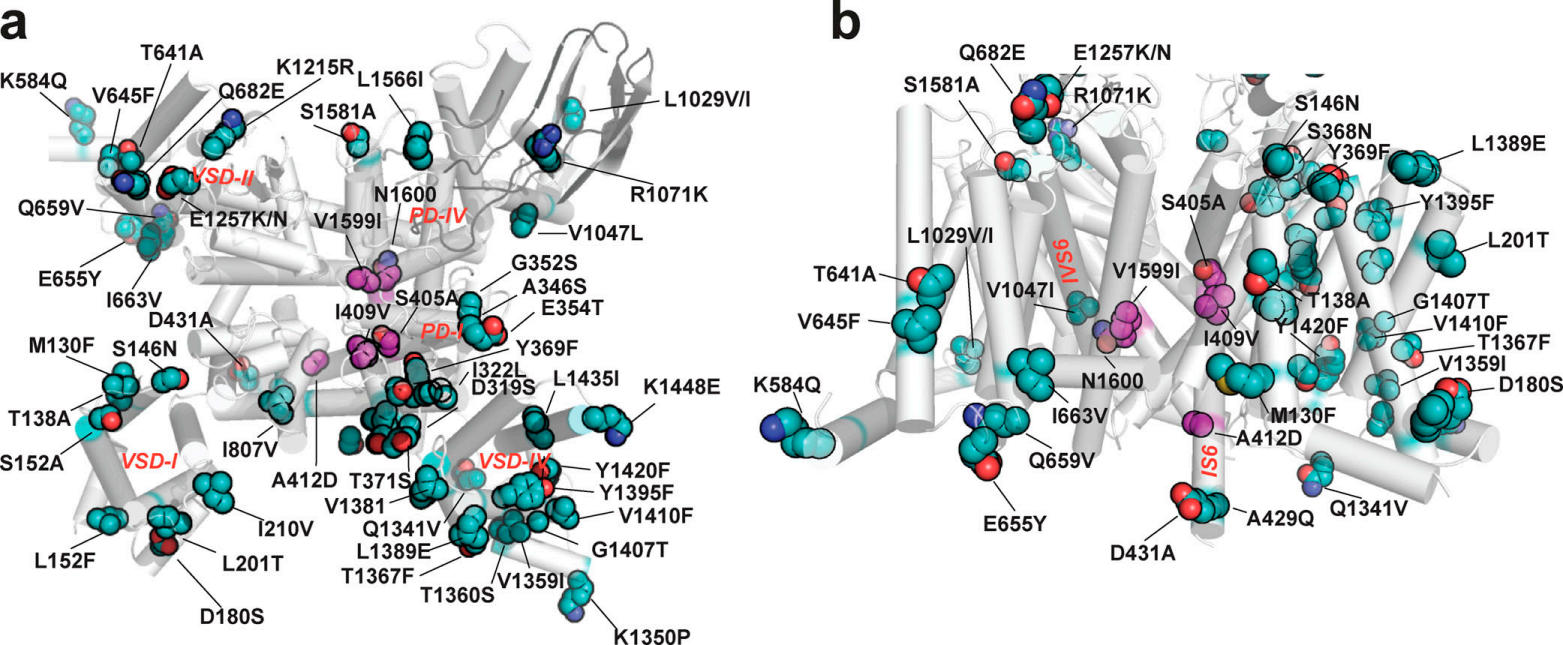
**Structural context for poison frog Na_V_ amino acid changes. (aandb)** Locations of poison frog Na_V_ amino acid variants reported here (cyan) and shared with Tarvin et al. (2016) (magenta). Variants are denoted human residue:residue number:frog variant using *Pt* Na_V_1.4 numbering from Fig. S1. Residues are mapped on human Na_V_1.4 (Protein Data Bank accession no. 6ADF; Pan et al., 2018). Na_V_1.4 (white).

Poison frogs in the genus *Phyllobates* (family Dendrobatidae) are the most well-known BTX carriers (Albuquerque et al., 1971; Santos et al., 2016; Albuquerque et al., 1973). A number of studies have identified amino acid substitutions hypothesized to contribute to poison frog Na_V_ BTX resistance (Tarvin et al., 2016; Márquez et al., 2019; Wang and Wang, 2017). We cloned poison frog Na_V_1.4 from the skeletal muscle from captivity-raised members of two representative poison frog species, one that carries high BTX levels in the wild, *Phyllobates terribilis* (*Pt* Na_V_1.4) (Tarvin et al., 2016; Daly et al., 1980; Myers et al., 1978), and one not known to carry BTX, *Dendrobates tinctorius* (*Dt* Na_V_1.4) (Daly et al., 1987). Consistent with evolutionary relationships between the two species (Tarvin et al., 2016; Márquez et al., 2019), *Pt* Na_V_1.4 and *Dt* Na_V_1.4 were highly similar to each other (∼95% amino acid identity) and other vertebrate Na_V_s (∼73% amino acid identity), and bore all Na_V_ hallmark features (Fig. S1). Importantly, their DIS6 and DIVS6 sequences were identical to those reported previously (Tarvin et al., 2016) with the remarkable absence in *Pt* Na_V_1.4 of the proposed BTX resistance mutation DIVS6 N→T (*Pt* Na_V_1.4 Asn1600, *Pt* Na_V_1.4 N1584T [rat numbering]; Wang and Wang, 2017; Fig. S1; Table S2). Genomic DNA sequencing covering the *Pt* Na_V_1.4 DIVS6 yielded nucleotide sequences identical to those obtained from cDNA and cross-validated the absence of the DIVS6 N→T substitution. These findings are consistent with the observation that the DIVS6 N1600T substitution has a very low frequency among *P. terribilis* (Márquez et al., 2019). Besides the prior reported amino acid variants (Tarvin et al., 2016), *Pt* Na_V_1.4 and *Dt* Na_V_1.4 differed from bird, human, and rat Na_V_1.4 at an additional 93 positions distributed throughout the channel (Fig. 2 and Table S2). Although we could readily sequence the *Pt* Na_V_1.4 and *Dt* Na_V_1.4 genes, both proved prone to recombination upon passage through *Escherichia coli*, rendering the native DNA sequences impossible to handle. To solve this problem, we redesigned the codon usage to preserve the amino acid sequence of both. These redesigned genes were well behaved and allowed us to conduct electrophysiological characterization of *Pt* Na_V_1.4 and *Dt* Na_V_1.4 in mammalian and amphibian expression systems.

Whole-cell patch-clamp electrophysiology of HEK293 cells transfected with *Pt* Na_V_1.4 and *Dt* Na_V_1.4 yielded voltage-dependent channels that matched the properties of *Pum* and *Hs* Na_V_1.4s (Fig. 1, e–h; Table 1; and Table S1). Strikingly, both poison frog Na_V_s had the same response to 10 µM BTX as *Pum* and *Hs* Na_V_1.4 (Fig. 1, e–h; ΔV_1/2 BTX_ = −30.0 ± 2.1 and −37.9 ± 2.0 mV for *Pt* Na_V_1.4 and *Dt* Na_V_1.4, respectively). Even though the expression levels of both frog channels were lower than those for the *Pitohui* or human channels, rendering their biophysical characterization less accurate, we found no evidence for Na_V_s in mock-transfected cells (Fig. 1 i and Table 1). We further examined the response of *Dt* Na_V_1.4 in a second system, CHO cells, and found similarly low, BTX-responsive Na_V_ currents that were absent from mock-transfected cells (Fig. S4, a, b, e, and f; and Table 1), giving confidence that the measured activity indeed arises from the frog Na_V_s. Hence, these results demonstrate that these poison frog channels are not resistant to BTX and rule out the possibility that the >90 amino acid differences between poison frog and human channels, including the previously proposed changes in DIS6 and DIVS6 (Tarvin et al., 2016), could confer BTX resistance. Notably, the response of the *Pt* Na_V_1.4 to BTX reveals a BTX sensitivity evoked by a BTX concentration (10 µM) that is well below that found in wild *P. terribilis* (∼170 µM; Myers et al., 1978).

Because expression of amphibian channels in an amphibian cell could provide a more native-like context, we also expressed *Pt* Na_V_1.4 and *Dt* Na_V_1.4 in *Xenopus* oocytes and examined their function by two-electrode voltage clamping. Both channels had biophysical parameters that matched those measured in mammalian cells (Fig. S4, g–j; and Table 1). Furthermore, BTX application caused the strong hallmark functional modification observed for all of the other channels we studied (Fig. 1; Fig. S3, a, b, d, and e; and Fig. S4, a–d), including voltage-dependent activation shifts comparable to those measured in mammalian cells (ΔV_1/2 BTX_ = −33.1 ± 3.2 and −30.0 ± 2.1 and −37.8 ± 6.1 and −37.9 ± 2.0 mV for *Pt* Na_V_1.4 and *Dt* Na_V_1.4 expressed in *Xenopus* oocytes and HEK293 cells, respectively). The shift was more complete in oocytes (cf. Fig. 1, f and h; and Fig. S4, h and j). This result likely originates from the fact that, for technical reasons, due to the large extracellular solution volumes used in the oocyte experiments that would require prohibitively large quantities of BTX, this toxin was injected into the oocytes rather than applied by bath application as it was for mammalian cells. Our observation that Na_V_s from two classes of BTX-carrying animals, *Pitohui* and *P. terribilis,* are not BTX resistant challenges the idea that Na_V_ mutation is the BTX autoresistance strategy as suggested for poison frogs such as *P. terribilis* (Tarvin et al., 2016; Wang and Wang, 2017).

### DIVS6 N→T mutation fails to confer BTX resistance to poison frog Na_V_s

**Figure 3.**
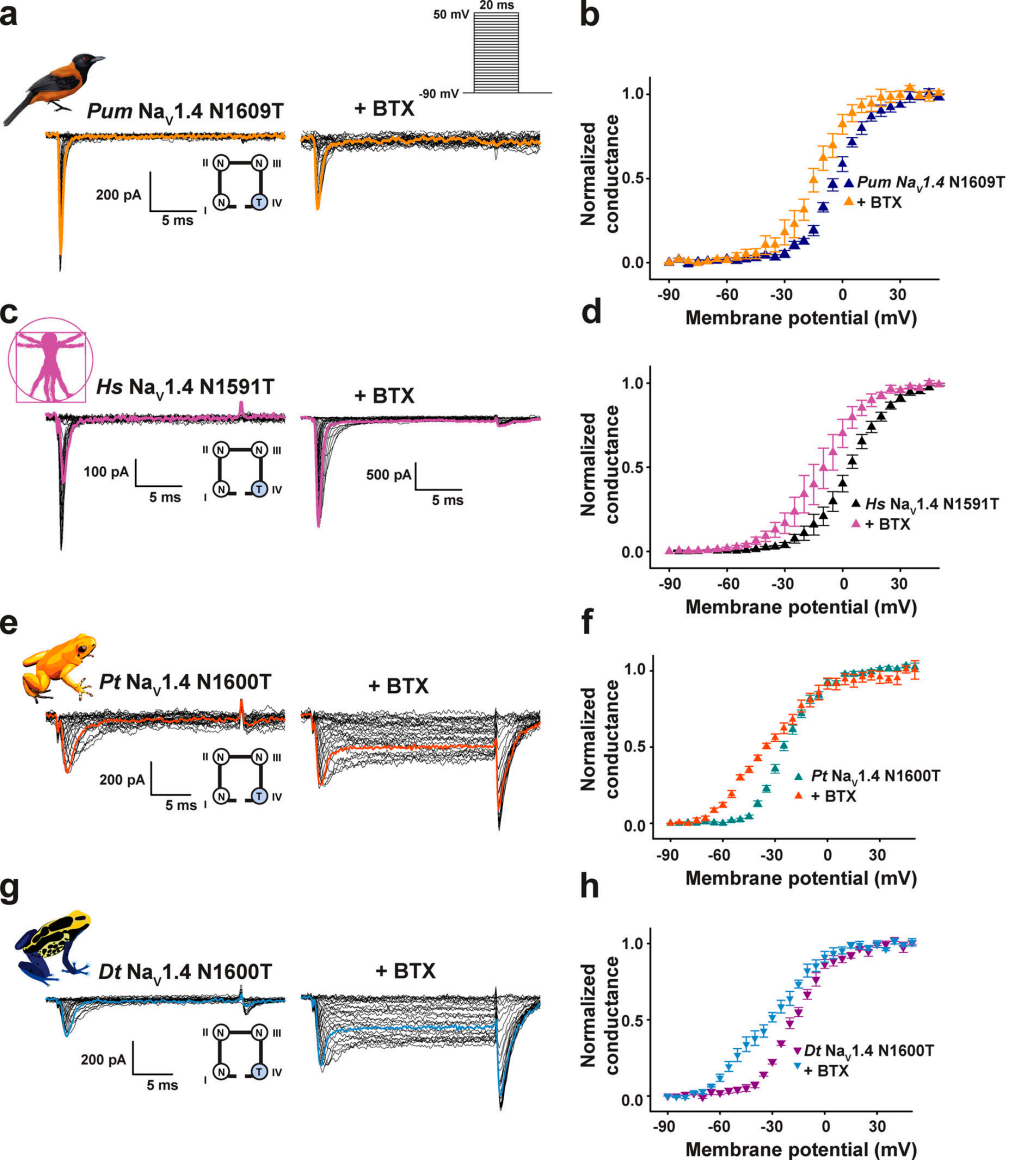
**DIVS6 N→T mutation reduces BTX sensitivity of *Pitohui* and human but not poison frog Na_V_1.4s.(a, c, e, and g)** Exemplar current recordings for *Pum* Na_V_1.4 N1609T (a), *Hs* Na_V_1.4 N1591T (c), *Pt* Na_V_1.4 N1600T (e), and *Dt* Na_V_1.4 N1600T (g) expressed in HEK293 cells in the absence (left) or presence (right) of 10 µM BTX. Trace at 0 mV is highlighted in each panel. Currents were evoked with the shown multistep depolarization protocol (inset in a). Cartoon shows a diagram of the identities of the S6 Asn for each construct. **(b, d, f, and h)** G-V relationships for *Pum* Na_V_1.4 N1609T (dark blue triangles), +BTX (orange triangles; b), *Hs* Na_V_1.4 N1591T (black triangles), +BTX (magenta triangles; d), *Pt* Na_V_1.4 N1600T (teal triangles), +BTX (dark orange triangles; f), and *Dt* Na_V_1.4 N1600T (magenta downward triangles), +BTX (cyan downward triangles; h) in the presence or absence of 10 µM BTX.

Because the DIVS6 N→T mutation was absent from Na_V_s of BTX-bearing species, we wondered whether the observation that DIVS6 N→T could confer BTX resistance to rat Na_V_1.4 (Wang and Wang, 2017) was impacted by the >90 amino acid differences between poison frog and mammalian Na_V_s (Fig. 2 and Table S2). Therefore, we placed the DIVS6 N→T mutation in poison bird, human, and poison frog Na_V_1.4s (*Pum* Na_V_1.4 N1609T, *Hs* Na_V_1.4 N1591T, *Pt* Na_V_1.4 N1600T, and *Dt* Na_V_1.4 N6100T) and measured its effects on channel function and BTX sensitivity. Consistent with studies of rat Na_V_1.4 DIVS6 N→T (Wang and Wang, 2017), DIVS6 N→T eliminated the ability of BTX to block inactivation and induce large tail currents in *Pum* Na_V_1.4 and *Hs* Na_V_1.4 (Fig. 3, a–d). Nevertheless, the bird and human Na_V_1.4s were not rendered completely BTX resistant. Application of 10 µM BTX shifted the voltage-dependent activation of both channels, making them more easily opened by voltage (ΔV_1/2 BTX_ = −11.4 ± 3.2 and −14.2 ± 5.5 mV for *Pum* Na_V_1.4 N1609T and *Hs* Na_V_1.4 N1591T, respectively; Fig. 3, b and d; and Table 1). Furthermore, the BTX-induced double Boltzmann was lost (Fig. 3, b and d), suggesting an enhanced BTX affinity. Due to its limited effectiveness in blocking the effects of BTX in the bird and human channels, we revisited the consequences of the DIVS6 N→T mutation in *Rn* Na_V_1.4. Similar to the bird and human channel results, DIVS6 N→T reduced but did not eliminate *Rn* Na_V_1.4 BTX sensitivity (Fig. S5, a–d). Application of 10 µM BTX shifted the voltage-dependent activation of both *Rn* Na_V_1.4 and *Rn* Na_V_1.4 N1584T, making them more easily opened by voltage (ΔV_1/2 BTX_ = −45.6 ± 0.4 and −40.3 ± 2.8 mV for *Rn* Na_V_1.4 and *Rn* Na_V_1.4 N1584T, respectively; Fig. S5, b and d; and Table 1). Thus, DIVS6 N→T was unable to mitigate the effects of BTX completely in any Na_V_ orthologue.

In all three contexts, DIVS6 N→T also affected channel biophysical properties (Fig. S5, e–g; Fig. S6, a–h; Tables 1 and 2; and Table S1). DIVS6 N→T rendered *Pum* Na_V_1.4, *Hs* Na_V_1.4, and *Rn* Na_V_1.4 more difficult to open, shifting the activation voltage dependence to depolarizing potentials (ΔV_1/2_ = +20.5 ± 1.5, +16.9 ± 2.7, and +18.0 ± 0.6 mV for *Pum* Na_V_1.4 N1609T, *Hs* Na_V_1.4 N1591T, and *Rn* Na_V_1.4 N1584T, respectively; Fig. S5 e; Fig. S6, b and f; and Tables 1 and 2), and made the channels easier to inactivate, shifting the voltage dependence of steady-state inactivation toward hyperpolarizing potentials (ΔV_1/2 inact_ = −10.3 ± 2.1, −9.8 ± 1.2, and −27.0 ± 0.4 mV for *Pum* Na_V_1.4 N1609T, *Hs* Na_V_1.4 N1591T, and *Rn* Na_V_1.4 N1584T, respectively; Fig. S5 f; Fig. S6, c and g; Table 2; and Table S1). Furthermore, DIVS6 N→T diminished *Pum* Na_V_1.4 N1609T, *Hs* Na_V_1.4 N1591T, and *Rn* Na_V_1.4 N1584T current densities by 79%, 55%, and 90%, respectively (Fig. S5, a, c, and g; Fig. S6, a, d, e, and h; and Tables 1 and 2). Thus, the DIVS6 N→T change incurs a substantial functional cost.

**Table 2.** Comparison of the functional effects of S6 mutations

Channel	BTXresistant?	Activation shift? ΔV_1/2 act_(mV)	Inactivation shift? ΔV_1/2 inact_(mV)	Decrease in current density? (fold change)
*Pum* Na_V_1.4 N432T (DI)	No	No	No	No
*Pum* Na_V_1.4 N830T (DII)	No	No	Yes; −10.8 ± 1.6	Yes; 14.8
*Pum* Na_V_1.4 N1306T (DIII)	No	No	No	No
*Pum* Na_V_1.4 N1609T (DIV)	Yes	Yes; +20.5 ± 1.5	Yes; −10.3 ± 2.1	Yes; 4.9
*Pum* Na_V_1.4 N1609A (DIV)	No	Yes; +19.6 ± 1.6	Yes; −13.4 ± 1.6	Yes; 4.1
*Hs* Na_V_1.4 N1591T (DIV)	Yes	Yes; +16.9 ± 2.7	Yes; −9.8 ± 1.2	Yes; 2.2
*Rn* Na_V_1.4 N1584T (DIV)	Yes	Yes; +18.0 ± 0.6	Yes; −27.0 ± 0.4	Yes; 10.3
*Pt* Na_V_1.4 (N1600T) (DIV)	No	No	Yes; −7.7 ± 0.9	No*
*Dt* Na_V_1.4 (N1600T) (DIV)	No	No	Yes; −9.8 ± 1.9	No*

Data represent measurements in transfected HEK293 cells. *, substantial current density loss in *Xenopus* oocytes. ΔV_1/2_ _act_ = V_1/2, act mutant_ -V_1/2, act WT_. ΔV_1/2 inact = _V_1/2, inact mutant_ -V_1/2, inact WT._

To probe the DIVS6 Asn site further, we examined the consequences of mutation to alanine in *Pum* Na_V_1.4. *Pum* Na_V_1.4 N1609A phenocopied the biophysical changes measured for N1609T, producing channels that were more difficult to open (ΔV_1/2_ = +19.6 ± 1.6 mV) and easier to inactivate (ΔV_1/2 inact_ = −13.4 ± 1.6 mV) and that had current density reduced by 76% (Fig. S6, a–d; Tables 1 and 2; and Table S1), in agreement with the reduced channel activity reported for the corresponding *Rn* Na_V_1.4 mutant (Wang et al., 1997; Sheets et al., 2015). These biophysical changes match those of the BTX-resistant *Pum* Na_V_1.4 N1609T; however, *Pum* Na_V_1.4 N1609A retained all of the classical BTX responses such as reduction of inactivation, enhanced tail current, and a leftward shift of the activation voltage dependence (Fig. S6, i and j; and Tables 1 and 2). The failure of the N1609A to diminish BTX sensitivity shows that the reduction of BTX sensitivity in *Pum* Na_V_1.4 N1609T, *Hs* Na_V_1.4 N1591T, and *Rn* Na_V_1.4 N1584T is a specific effect of the threonine mutation and not a consequence of the changes in channel biophysical properties or current density reduction (Table 2).

To our surprise, placing DIVS6 N→T in both poison frog Na_V_1.4s failed to blunt the effects of BTX on channel activation and inactivation (Fig. 3, e–h; ΔV_1/2 BTX_ = −30.0 ± 2.1 and −30.8 ± 2.5 mV for *Pt* Na_V_1.4 and *Pt* Na_V_1.4 N1600T, respectively, and −37.9 ± 2.0 and −37.8 ± 1.8 for *Dt* Na_V_1.4 and *Dt* Na_V_1.4 N1600T, respectively). Similar results were obtained for *Dt* Na_V_1.4 N1600T measured in a second expression system, CHO cells (Fig. S4, c, d, and f). Hence, even though the DIVS6 N→T change reduces *Pum* Na_V_1.4, *Hs* Na_V_1.4, and *Rn* Na_V_1.4 BTX responses (Fig. 3, a–d; and Fig. S5, a–d), this same change fails to affect the BTX sensitivity of poison frog Na_V_s. Unlike its effects in *Pum, Hs,* and *Rn* Na_V_s, DIVS6 N→T did not cause major changes in poison frog Na_V_ biophysical properties, shifting only the inactivation voltage dependence by −10 mV while leaving the activation voltage dependence and current density unchanged (Fig. S7, a–c; Tables 1 and 2; and Table S1). Additionally, expression of *Pt* Na_V_1.4 N1600T and *Dt* Na_V_1.4 N1600T in *Xenopus* oocytes revealed dramatic reductions in channel activity (Fig. S7, d–h). This ∼10-fold reduction in current amplitude prevented measurement of channel biophysical properties and BTX responses, especially because in the case of the latter, the intracellular injection of BTX necessary for the oocyte experiments induces a leak current that is similar to the amplitude of the N1600T mutants. Nevertheless, these results reveal that DIVS6 N→T is detrimental to function and may interfere with channel folding and maturation in a manner that is accentuated at lower temperatures, such as those used to store the oocytes. This context-dependent loss of function indicates that the DIVS6 N→T variant exacts a functional cost that, together with its ineffectiveness in endowing poison frog Na_V_s with BTX resistance, challenges the idea that DIVS6 N→T could serve as an effective BTX autoresistance mechanism.

### Cost of the conserved N→T mutation is context dependent

**Figure 4.**
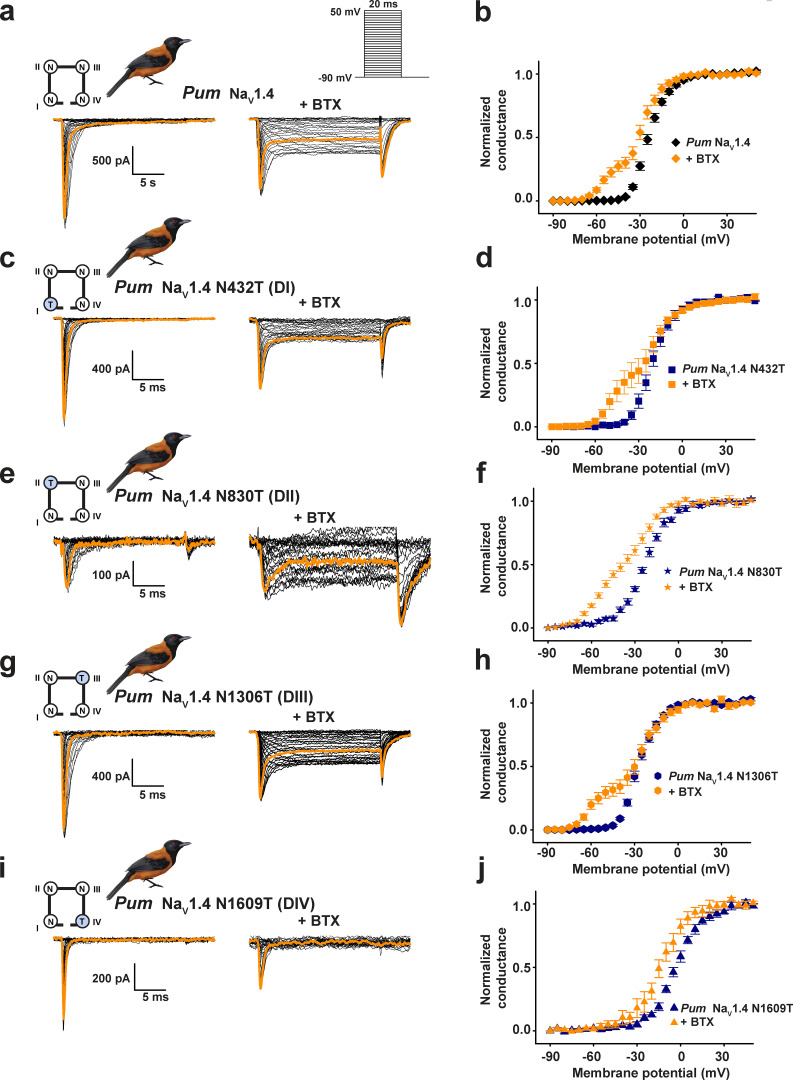
**Sodium channel modulation by BTX is associated with an asymmetry in the inner pore.(a, c, e, g, and i)** Exemplar current recordings for *Pum* Na_V_1.4 (a), *Pum* Na_V_1.4 N432T (c), *Pum* Na_V_1.4 N830T (e), *Pum* Na_V_1.4 N1306T (g), and *Pum* Na_V_1.4 N1609T (i) expressed in HEK293 cells in the absence (left) or presence (right) of 10 µM BTX. Trace at 0 mV is highlighted in each panel. Currents were evoked with the shown multistep depolarization protocol (inset in a). Cartoon shows a diagram of the identities of the S6 Asn for each construct. **(b, d, f, h, and j)** G-V relationships in the presence or absence of 10 µM BTX for *Pum* Na_V_1.4 (black diamonds), +BTX (orange diamonds; b), *Pum* Na_V_1.4 N432T (dark blue squares), +BTX (orange squares; d), *Pum* Na_V_1.4 N830T (dark blue stars), +BTX (orange stars; f), *Pum* Na_V_1.4 N1306T (dark blue hexagons), +BTX (orange hexagons; h), and *Pum* Na_V_1.4 N1609T (dark blue triangles), +BTX (orange triangles; j). Data in a and b are from Fig. 1, a and b.

The varied outcomes of DIVS6 N→T on BTX sensitivity among the poison bird, human, rat, and poison frog Na_V_s highlight the importance of context in determining the functional consequences of mutations. Because the equivalent residue is conserved in all four S6 helices (Figs. S1 and S2), we systematically introduced S6 N→T into each of the *Pum* Na_V_1.4 S6 segments and measured channel properties and BTX responses to investigate the question of context-dependent effects further (Fig. 4). Whole-cell patch-clamp recordings from HEK293 cells transfected with these mutant channels revealed clear, domain-specific differences. Contrasting the effect of DIVS6 N→T (Fig. S6 b), voltage-dependent activation of channels having the N→T mutation in DI, DII, or DIII (*Pum* Na_V_1.4 N432T [DI], *Pum* Na_V_1.4 N830T [DII], and *Pum* Na_V_1.4 N1306T [DIII]) was unchanged relative to WT (V_1/2_ = −20.2 ± 1.9, −22.4 ± 1.0, −26.7 ± 1.0, and −23.4 ± 1.0 mV for *Pum* Na_V_1.4 N432T [DI], *Pum* Na_V_1.4 N830T [DII], *Pum* Na_V_1.4 N1306T [DIII], and *Pum* Na_V_1.4, respectively; Fig. 4, a–j; Fig. S8, a and b; and Tables 1 and 2). By contrast, we found varied effects on steady-state inactivation. DI and DIII changes showed WT-like behavior, whereas the DII mutant had an ∼10-mV hyperpolarizing shift (V_1/2 inact_ = −61.2 ± 1.6, −75.0 ± 0.9, −65.3 ± 1.0, and −64.2 ± 1.3 mV for *Pum* Na_V_1.4 N432T [DI], *Pum* Na_V_1.4 N830T [DII], *Pum* Na_V_1.4 N1306T [DIII], and *Pum* Na_V_1.4, respectively; Fig. S8 c, Table 2, and Table S1). All three had strong BTX responses similar to WT (ΔV_1/2 BTX_ = −30.8 ± 2.1, −31.6 ± 2.0, −36.9 ± 1.4, and −33.6 ± 1.2 mV for *Pum* Na_V_1.4 N432T [DI], *Pum* Na_V_1.4 N830T [DII], *Pum* Na_V_1.4 N1306T [DIII], and *Pum* Na_V_1.4, respectively; Fig. 4 and Tables 1 and 2). Thus, the only site where the conserved S6 N→T change affects BTX responses is in DIVS6, in line with its proposed contribution to the BTX binding site (Wang and Wang, 2017).

As with the biophysical changes, the effects on current density from placing the N→T change in different channel domains were not uniform. The DIS6 and DIIIS6 N→T mutants had current densities matching WT (Fig. 4, a, c, and g; Fig. S8, a and d; and Tables 1 and 2), whereas, DIIS6 N→T lowered the current density and was more detrimental to channel activity than DIVS6 N1609T or N1609A (Fig. 4, a and e; Fig. S8, a and d; and Tables 1 and 2). Together, these data show that there is no correlation between changes in channel biophysical properties and the acquisition of BTX resistance and are in line with the results from DIVS6 N→T and N→A mutants (Figs. 3, S6, and S7, and Table 2).

Consideration of the conserved S6 asparagine structural locale provides insight into the context-dependent effects. The two S6 sites where N→T has no impact on channel biophysics, BTX responses, or current density, DIS6 and DIIIS6, occupy positions that are partially exposed to the channel inner pore (Fig. S8, e and f). By contrast, the two positions that affect channel biophysics and current density, DIIS6 and DIVS6, interact with the S4–S5 linkers (Pan et al., 2018; Fig. S8, e and f), and altering these buried sites comes with substantial functional costs. Hence, DIVS6 N→T carries major disadvantages for protecting animals such as *Pitohui* and poison frogs against BTX autointoxication.

### Toxin-free poison frogs have BTX- and STX-sensitive Na_V_s but are resistant to both toxins

The surprising observation that Na_V_s from BTX-carrying birds and frogs remain BTX sensitive raised the question whether the species from which we cloned the channels were actually BTX resistant. Because of difficulties in obtaining live animals, we were unable to investigate *Pitohui* BTX resistance. Captivity-raised poison frogs lack BTX because this toxin is acquired in the wild from their diet (Daly et al., 1994b; Daly et al., 1994a). Thus, it was possible that the toxin-free poison frogs used to clone Na_V_s were not BTX resistant due to the absence of selective pressure from the toxin, a possibility underscored by the high functional cost of DIVS6 N→T (Fig. 3, e–h; Fig. S7; Tables 1 and 2; and Table S1). To test whether captivity-raised poison frogs were BTX resistant, we conducted a series of toxin challenge experiments using five different frog species: two nonpoisonous frogs (*Xenopus* and *Polypedates* *leucomystax*), two captivity-raised dendrobatid poison frogs that carry alkaloid toxins in the wild (*P. terribilis*, BTX; and *D. tinctorius*, HTX and PTX), and an unrelated captivity-raised Malagasy poison frog that carries PTX rather than BTX in the wild (*M. aurantiaca*) and that represents an independent evolutionary origin of chemical defenses (Daly et al., 2005; Daly et al., 2008; Garraffo et al., 1993). We challenged these animals with three different toxins that target Na_V_s: BTX and two guanidinium toxins that act by a pore-blocking mechanism, STX (Thottumkara et al., 2014; Durán-Riveroll and Cembella, 2017) and TTX (Durán-Riveroll and Cembella, 2017).

**Figure 5.**
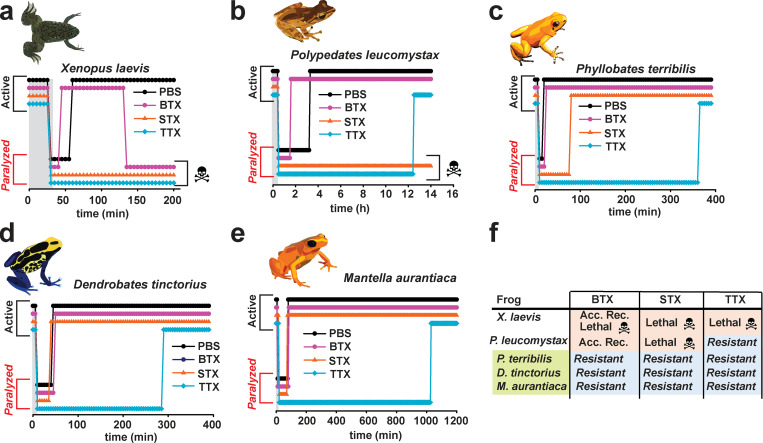
**Captivity-raised poison frogs are resistant to BTX and STX. (a–e)** Challenge experiments for *Xenopus* (a), *P. leucomystax* (b), *P. terribilis* (c), *D. tinctorius* (d), and *M. aurantiaca* (e) with PBS (black circles), BTX (magenta circles), STX (orange triangles), or TTX (cyan diamond) injection. Gray area shows the period of anesthesia application. Active and paralyzed states of the frogs are indicated. **(f)** Summary of the sensitivity of the indicated species to BTX, STX, and TTX. Acc. Rec., accelerated recovery from anesthesia; Resistant, no toxin-induced death.

We assessed the duration of recovery from anesthesia-induced paralysis after intramuscular injection of each toxin at 20 times the lethal dose based on values for mice (LD_50_) by monitoring how long it took the frog to show clear motor activity relative to injection of a PBS control. After BTX injection, *Xenopus* and *P. leucomystax* displayed an accelerated recovery from anesthesia that was at least two times faster than that with PBS injections (PBS and BTX recovery times: 29 ± 1 min and 15 ± 5 min and 169 ± 12 min and 70 ± 20 min for *Xenopus* and *Phyllobates leucomystax*, respectively; Fig. 5, a, b, and f; and Table S3). After the initial recovery, BTX was ultimately lethal to *Xenopus* (Fig. 5, a and f). By contrast, BTX injection did not change the anesthesia recovery time or kill any of the poison frogs, regardless of whether they carry BTX in the wild (*P. terribilis*) or are naturally BTX free but harbor other alkaloid toxins (*D. tinctorius, M. aurantiaca*; Fig. 5, c–f; and Table S3). Reponses to STX also revealed differences between nonpoisonous and poisonous frogs. STX injection was lethal to *Xenopus* and *P. leucomystax* (Fig. 5, a, b, and f), whereas all three poison frogs fully recovered from anesthesia after STX injections (Fig. 5, c–f). TTX was lethal to *Xenopus* (Fig. 5, a and f). Although TTX caused extended paralysis in all other tested frogs, it was not lethal (Fig. 5, a–f; and Table S3). Thus, all tested poisonous frogs showed resistance to all three toxins, whereas nonpoisonous frogs were vulnerable to either BTX and STX (*P. leucomystax*) or all three toxins (*Xenopus*; Fig. 5 f).

**Figure 6.**
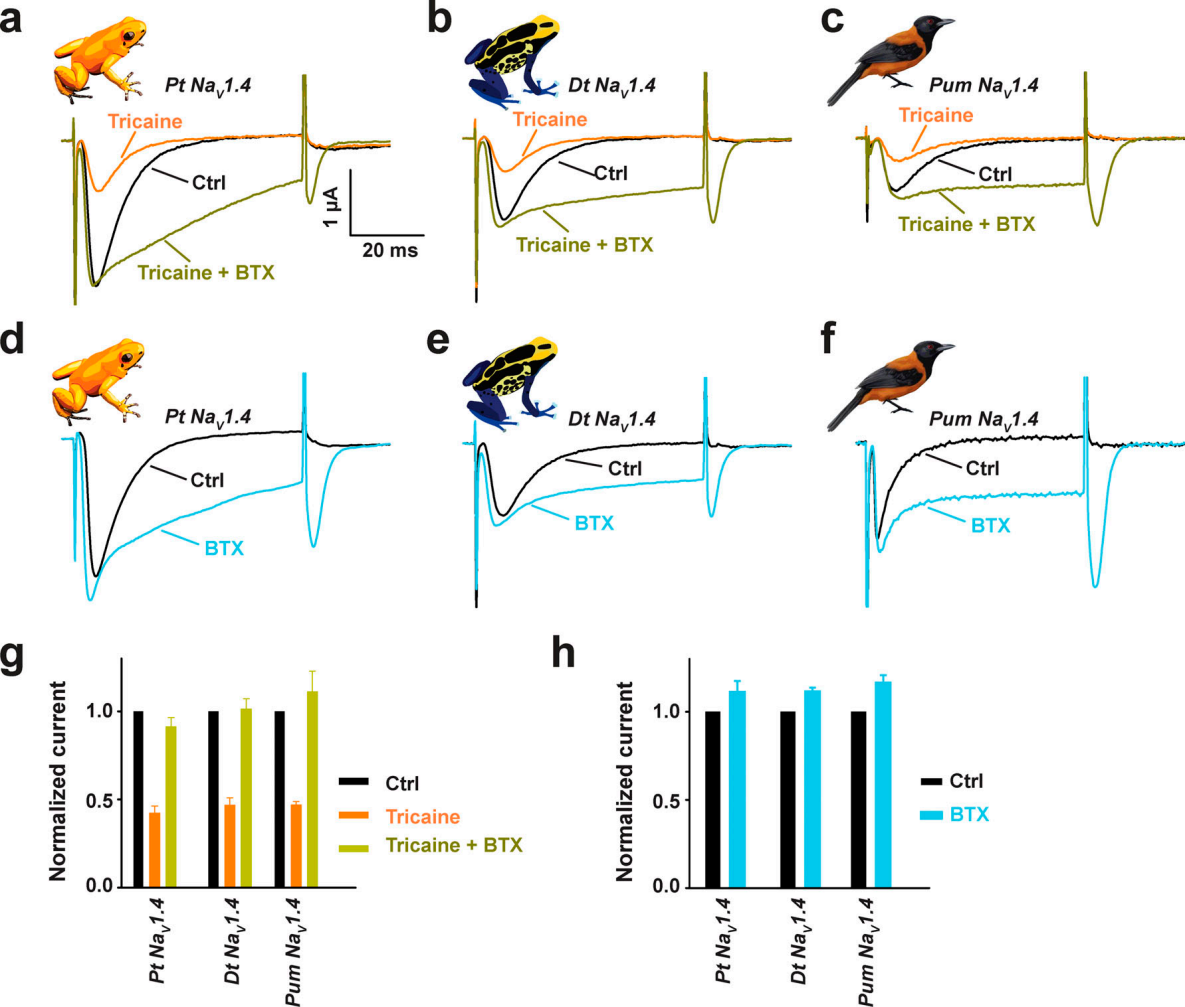
**BTX competes with anesthetic agent tricaine in Na_V_s from poisonous species. (a–c)** Exemplar two electrode voltage clamp (TEVC) recordings at 0 mV in control (black), after 0.5 mM tricaine application (orange), and after BTX injection (dark green) into the same *Xenopus* oocytes expressing *Pt* Na_V_1.4 (a), *Dt* Na_V_1.4 (b), and *Pum* Na_V_1.4 (c). BTX injection was performed after tricaine block of sodium current, and the recordings of the BTX effect were made while the oocyte was still exposed to tricaine. **(d–f)** Exemplar TEVC recordings at 0 mV before (black) or after BTX injection (light blue) into the same *Xenopus* oocytes expressing Na_V_1.4 from the indicated poisonous species. **(g and h)** Average peak current amplitudes normalized to the corresponding control peak current amplitude for tricaine and BTX (g) and BTX alone (h).

The striking differences in BTX-induced accelerated recovery from anesthesia between the nonpoisonous and poisonous species was unexpected. Studies of eukaryotic and prokaryotic Na_V_s suggest that BTX and local anesthetics, such as the tricaine used for frog anesthesia, have overlapping binding sites within the channel pore (Finol-Urdaneta et al., 2019; Wang and Wang, 1992; Wang et al., 1994; Wang and Wang, 1994). We considered that the differences in BTX-dependent accelerated recovery from anesthesia were a physiological manifestation of this molecular competition and indicated that BTX was engaging the target channels in nonpoisonous frogs but not in poisonous frogs. Hence, we tested whether tricaine and BTX produced competing effects on *Pt* Na_V_1.4 and *Dt* Na_V_1. 4. Consistent with its anesthetic effects on the frogs, 0.5 mM tricaine inhibited both poison frog Na_V_s and had similar effects on the *Pum* Na_V_1.4 control (Fig. 6, a–c and g). Subsequent BTX injection into the same tricaine-treated oocytes caused complete relief of the tricaine block (Fig. 6, a–c and g), in line with a direct competition between tricaine and BTX. By contrast, in the absence of tricaine, this dramatic BTX-induced increase in peak current was absent (Fig. 6, d–f and h). These data demonstrate that the poison frog Na_V_s are competent for BTX–tricaine competition. Hence, the differences in BTX-induced accelerated recovery from anesthesia reflect the direct competition of the two compounds on the channel in the nonpoisonous frogs and suggest that the poisonous frogs have a means to prevent BTX from engaging their Na_V_s.

**Figure 7.**
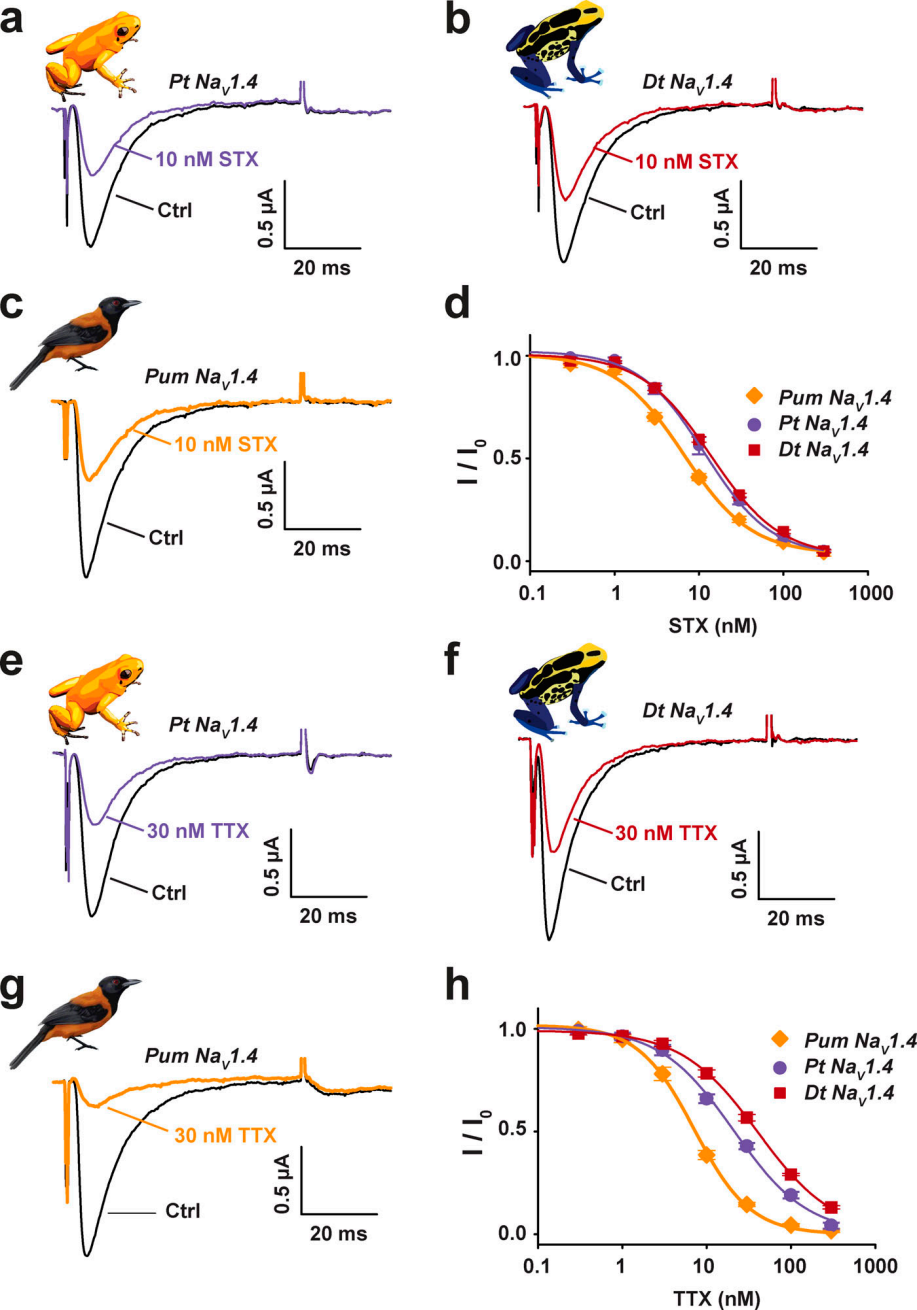
**Na_V_1.4s from poisonous animals are STX and TTX sensitive. (a–c)** Exemplar two-electrode voltage-clamp (TEVC) recordings at 0 mV before (black) and after 10 nM STX application to *Xenopus* oocytes expressing *Pt* Na_V_1.4 (purple; a), *Dt* Na_V_1.4 (red; b), or *Pum* Na_V_1.4 (orange; c). **(d)** STX dose–response curves for *Pt* Na_V_1.4 (purple circles), *Dt* Na_V_1.4 (red squares), and *Pum* Na_V_1.4 (orange diamonds). Curves show fits to the Hill equation. IC_50_ = 12.6 ± 1.4 nM, 14.6 ± 0.6 nM, and 7.3 ± 0.5 nM for *Pt* Na_V_1.4, *Dt* Na_V_1.4, and *Pum* Na_V_1.4, respectively. Error bars are SEM. *n* = 4. **(e–g)** Exemplar TEVC recordings at 0 mV before (black) and after 30 nM TTX application to *Xenopus* oocytes expressing *Pt* Na_V_1.4 (purple; e), *Dt* Na_V_1.4 (red; f), or *Pum* Na_V_1.4 (orange; g). **(h)** TTX dose–response curves for *Pt* Na_V_1.4 (purple circles), *Dt* Na_V_1.4 (red squares), and *Pum* Na_V_1.4 (orange diamonds). Curves show fits to the Hill equation. IC_50_ = 21.3 ± 1.0 nM, 40.8 ± 1.8 nM, and 6.2 ± 0.4 nM for *Pt* Na_V_1.4, *Dt* Na_V_1.4, and *Pum* Na_V_1.4, respectively. Error bars are SEM. *n* = 5–6.

The poison frog STX resistance we observed could be explained by a lack of Na_V_ sensitivity to this toxin. To test this possibility, we compared the responses of *Pt* Na_V_1.4, *Dt* Na_V_1.4, and *Pum* Na_V_1.4 as a control to STX and TTX because the former had minimal effect on the poison frogs, whereas the latter caused potent paralysis (Fig. 5 and Table S3). Extracellular application of increasing STX concentrations inhibited all three Na_V_s with a nanomolar response that matched that of other Na_V_s (Thomas-Tran and Du Bois, 2016; Thottumkara et al., 2014; Andresen and Du Bois, 2009; IC_50_ = 12.6 ± 1.4 nM, 14.6 ± 0.6 nM, and 7.3 ± 0.5 nM for *Pt* Na_V_1.4, *Dt* Na_V_1.4, and *Pum* Na_V_1.4, respectively; Fig. 7, a–d). *Pt* Na_V_1.4 and *Dt* Na_V_1.4 and the control *Pum* Na_V_1.4 also had nanomolar TTX responses, similar to *Hs* Na_V_1.4 (Chahine et al., 1994; IC_50_ = 21.3 ± 1.0 nM, 40.8 ± 1.8 nM, and 6.2 ± 0.4 nM for *Pt* Na_V_1.4, *Dt* Na_V_1.4, and *Pum* Na_V_1.4, respectively; Fig. 7, e–h). Thus, the ability of the poison frogs to resist STX does not arise from their Na_V_s having some unusual resistance to the toxin (Figs. 5 and 7 d and Table S3). The resistance of poison frogs to the effects of BTX and STX contrasts with the effects of TTX and is not consistent with the high sensitivity of their Na_V_1.4s to all three toxins. These findings suggest that mechanisms such as toxin sequestration may prevent BTX and STX from reaching their target Na_V_s.

### An amphibian “toxin sponge” protein protects Na_V_s from toxin action

**Figure 8.**
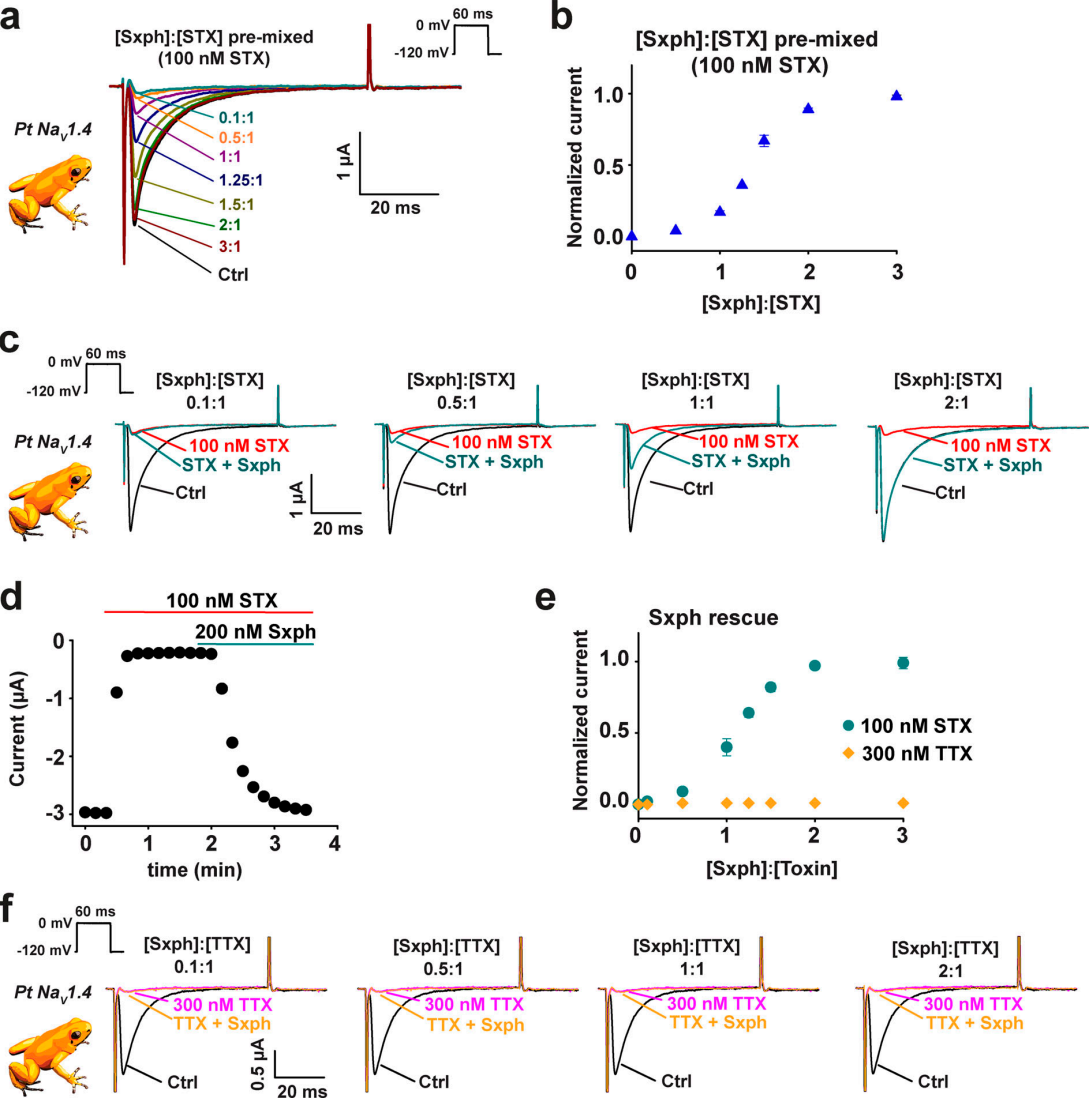
**Sxph rescues *Pt* Na_V_1.4 from STX block. (a)** Exemplar two-electrode voltage-clamp (TEVC) recordings of *Pt* Na_V_1.4 expressed in *Xenopus* oocytes in the presence of 100 nM STX and [Sxph]:[STX] in the indicated molar ratios. Ctrl (black) shows response in the absence of STX. Inset shows the stimulation protocol. **(b)** [Sxph]:[STX] dose response from a. **(c)** Exemplar TEVC recordings of *Pt* Na_V_1.4 before (black) and after (red) application of 100 nM STX and then after application of Sxph at the indicated [Sxph]:[STX] molar ratio (blue-green). Inset shows the protocol. **(d)** Exemplar TEVC time course showing *Pt* Na_V_1.4 peak currents from c after application of 100 nM STX (red bar) and 200 nM Sxph (blue-green bar). **(e)** [Sxph]:[toxin] dose response for 100 nM STX (blue-green circles) and 300 nM TTX (orange diamonds). Normalized current in b and e was determined by I = (I_Sxph_ − I_Toxin_)/(I_ctrl_ − I_Toxin_), where I_Sxph_ is the current after application of Sxph:STX mixtures in b or after Sxph addition for e, I_Toxin_ is the current after STX or TTX application, and I_Ctrl_ is the basal current. **(f)** Exemplar TEVC recordings of *Pt* Na_V_1.4 before (black) and after (magenta) application of 300 nM TTX and then after application of Sxph at the indicated [Sxph]:[TTX] molar ratio (orange). Inset shows the protocol. For all experiments, *n* = 5, and error bars indicate SEM.

**Figure S9.**
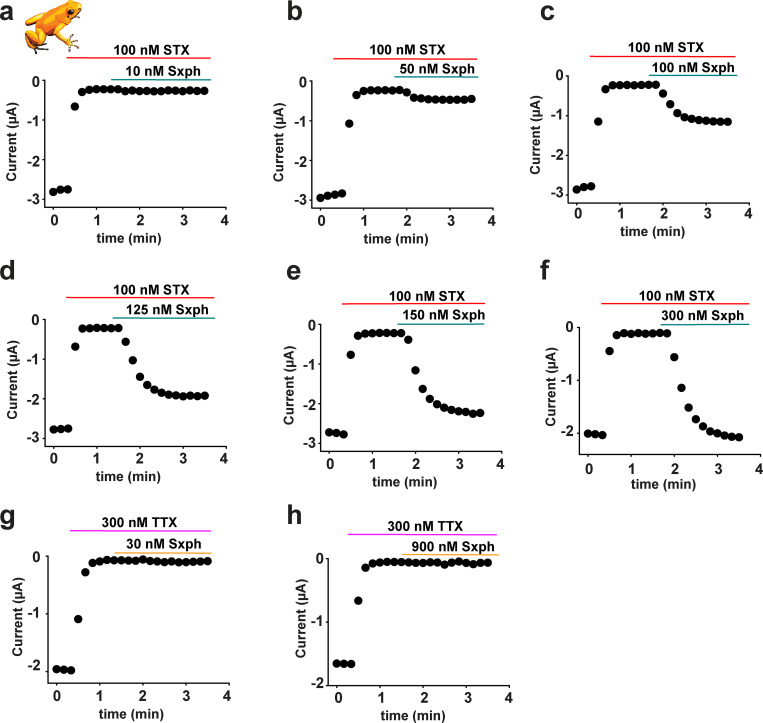
**Sxph reverses STX block of *Pt* Na_V_1.4. (a–f)** Exemplar two-electrode voltage-clamp (TEVC) time courses showing *Pt* Na_V_1.4 peak currents after application of 100 nM STX (red bar) and the indicated concentrations of Sxph (blue-green bar). **(g and h)** Exemplar TEVC time courses showing *Pt* Na_V_1.4 peak currents after application of 300 nM TTX (magenta bar) and the indicated concentrations of Sxph (orange bar).

The toxin challenge experiments indicated that BTX and STX were unable to affect poison frog Na_V_s, even though the channels are perfectly sensitive to both toxins (Figs. 5 and 7 d, and Table S3). Although there is yet no known BTX binding protein, a high-affinity soluble 91 kD STX-binding protein from frog plasma, Sxph, has been well characterized and shown to bind STX but not TTX (Mahar et al., 1991; Yen et al., 2019; Llewellyn and Moczydlowski, 1994; Morabito and Moczydlowski, 1994). Hence, we asked whether Sxph, which has a *K*_d_ for STX that is comparable to that of the channel (Mahar et al., 1991; Doyle et al., 1982; Llewellyn and Moczydlowski, 1994), would be capable of protecting Na_V_s from the action of STX. Application of solutions preequilibrated with different Sxph:STX molar ratios showed that Sxph was able to protect completely *Pt* Na_V_ expressed in *Xenopus* oocytes from STX inhibition once the ratio reached 2:1 Sxph:STX (Fig. 8, a and b). Furthermore, application of Sxph to cells expressing *Pt* Na_V_s that had been preblocked with STX yielded the same result and demonstrated that Sxph is able to compete effectively with the channel for the toxin (Fig. 8, c–e; and Fig. S9, a–f). Importantly, Sxph had no effect against the related guanidinium toxin TTX (Fig. 8, e and f; and Fig. S9, g and h), a toxin that does not bind Sxph (Mahar et al., 1991). Hence, these experiments demonstrate that high-affinity toxin sponge proteins are able to prevent the actions of small-molecule toxins that target Na_V_s and lend further support to the idea that toxin sequestration mechanisms may act to protect poisonous animals from autointoxication.

## Discussion

Poisonous organisms that use toxins as defensive molecules must avoid autointoxication. Such resistance has been proposed to arise from three strategies: (1) acquisition of target protein toxin resistance mutations, (2) toxin sequestration, and (3) enhanced detoxification or elimination capacity (Almabruk et al., 2018; Arbuckle et al., 2017). Support for the first mechanism includes prominent examples of TTX-resistant Na_V_s in toxin-bearing species and their predators (Hanifin and Gilly, 2015; Jost et al., 2008; Geffeney et al., 2002; Geffeney et al., 2005; McGlothlin et al., 2016), STX-resistant Na_V_s in mollusks (Bricelj et al., 2005), and epibatidine-resistant nicotinic acetylcholine receptors in poison frogs (Tarvin et al., 2017). Because of these examples and the suggestion that the Na_V_ DIVS6 N→T mutation might confer BTX resistance to poison frogs (Wang and Wang, 2017), we expected to find toxin-resistant mutants in poison bird and frog Na_V_s. Instead, we found multiple lines of evidence demonstrating that Na_V_s from both poison birds and frogs are highly sensitive to BTX and lack the DIVS6 N→T change. Furthermore, even though the DIVS6 N→T mutation alters the BTX responses of bird, human, and rat Na_V_1.4s (Fig. 3, a–d; and Fig. S5, a–d), it failed to have any effect on the BTX sensitivity of poison frog Na_V_s (Fig. 3, e–h), a result that highlights the importance of vetting putative toxin resistance mutations in the context of the native channel (Tarvin et al., 2017).

How amino acid changes compensate for mutations that alter function is complex and can arise from effects at positions that are far apart in the protein structure (Thomas et al., 2010; Wang et al., 2002; Bigman and Levy, 2020; Tokuriki et al., 2008). There are >90 amino acid differences between the poison frog and human Na_V_1.4s (Fig. 2 and Table S2), and it is not obvious which variants in the frog Na_V_s suppress the ability of DIVS6 N→T to affect BTX responses. The importance of context is further evident from the fact that even though the Asn position is conserved in all four Na_V_ pore domain subunits, the functional consequences of the N→T change are domain dependent (Fig. 4 and Table 2). These factors, together with the absence of DIVS6 N→T in BTX-bearing birds and frogs (Figs. S1 and S2; Márquez et al., 2019; Tarvin et al., 2016) and its ineffectiveness in poison frog Na_V_s, rule out the target alteration hypothesis for BTX resistance.

Endowing a protein with a new function through mutation often incurs a cost, particularly with respect to protein stability (Wang et al., 2002; Bigman and Levy, 2020; Tokuriki et al., 2008). Our data show that the DIVS6 N→T change in bird, human, rat, and frog Na_V_s carries substantial functional costs that affect every aspect of channel function by inducing changes that render the channels more difficult to open and more readily inactivated and that reduce current density (Table 2), an effect that likely reflects stability penalties that impact channel biogenesis (Baroudi et al., 2002; Solé and Tamkun, 2020; Mercier et al., 2017). These severe pleiotropic functional consequences are in line with the role of this conserved Asn site in coupling the pore to the voltage sensor domain in Na_V_s (Sheets et al., 2015). Similar perturbations of Na_V_ inactivation and reduction of current levels have profound physiological consequences (Chen et al., 2002) and are linked to a variety of channelopathies (Loussouarn et al., 2016), underscoring the organism-level fitness problems incurred by changes in Na_V_ biophysical properties. These substantial fitness costs, as well as the inability of the DIVS6 N→T mutation to affect the BTX responses of poison frog Na_V_s, are consistent with the low frequency of this variant in *P. terribilis* (Márquez et al., 2019) and its absence from the BTX-bearing *P. aurotaenia* poison frog (Tarvin et al., 2016). Other studies of ion channel toxin resistance mutants have uncovered various degrees of functional costs that may be compensated by amino acid changes at additional sites in the channel (Lee et al., 2011; Tarvin et al., 2017). Hence, the effectiveness of developing a toxin-resistant channel via mutation is highly dependent on the cost for evolving this new function and the extent to which functional costs can be mitigated by additional changes.

Poison frogs lacking the Na_V_1.4 DIVS6 N→T change withstand BTX levels that affect nonpoisonous frogs (Fig. 5 and Table S3), in line with previous studies (Daly et al., 1980). In nonpoisonous frogs, we find clear in vivo physiological antagonism between the channel blocker, tricaine, and the channel opener, BTX. This result indicates that both compounds access their target Na_V_s. By contrast, this antagonism is absent in poison frogs (Fig. 5 and Table S3), even though it can occur at the molecular level of the channel (Fig. 6). Furthermore, the resistance of poison frogs to BTX and STX contrasts with the effects of TTX and is not consistent with the high sensitivity of their Na_V_1.4s to all three toxins (Fig. 1, e–h; Fig. 5; and Fig. 7). Together these observations suggest that poison frogs have a means to prevent BTX and STX engaging the target Na_V_s. It is notable that other frogs resist STX poisoning (Prinzmetal et al., 1932; Kao and Fuhrman, 1967; Mahar et al., 1991), and it is thought that the soluble STX-binding protein Sxph (Mahar et al., 1991; Arbuckle et al., 2017; Yen et al., 2019) acts as a toxin sponge to sequester and neutralize the lethal effects of this and possibly other neurotoxins (Mahar et al., 1991; Llewellyn et al., 1997; Arbuckle et al., 2017; Almabruk et al., 2018; Caty et al., 2019; O’Connell et al., 2021).

If BTX-bearing animals do not use BTX-resistant Na_V_s to avoid autointoxication, how do they survive? Apart from the absence of BTX-resistant Na_V_s, the diversity among >800 poison frog alkaloid toxins (Daly et al., 2005), the seasonal and geographical variation of these toxins, and their ability to affect multiple ion channels (Santos et al., 2016) pose major challenges for evolving toxin-resistant channels. Enhanced detoxification via metabolic toxin destruction would not be useful, because these poisonous organisms need to handle and store the toxins to deploy them against predators. By contrast, sequestration strategies not only offer a general means of toxin protection but also could act in pathways involved in safely transporting and concentrating toxins in key defensive organs such as the skin (Menon and Dumbacher, 2014). The fact that toxin-based chemical defense systems have evolved independently four times in neotropical poison frogs (Dendrobatids; Santos et al., 2016), in Malagasy poison frogs (Garraffo et al., 1993), and in multiple lineages of poisonous birds (including *Pitohui* and *Ifrita*; Dumbacher et al., 1992; Dumbacher et al., 2000) supports the idea that such general sequestration mechanisms may underlie toxin autoresistance. Furthermore, there are a number of examples of poison frogs (Tarvin et al., 2016; Márquez et al., 2019) and predators of toxic animals (Feldman et al., 2016) that lack toxin-resistant mutations, raising questions about the generality of the target-based mechanism. Although no BTX-binding proteins have yet been identified, high-affinity toxin-binding proteins for STX in frogs, Sxph (Llewellyn et al., 1997; Mahar et al., 1991; Yen et al., 2019), and STX and TTX in pufferfish, pufferfish STX- and TTX-binding protein, PSTBP (Yotsu-Yamashita et al., 2001; Yotsu-Yamashita et al., 2010), are known and have been proposed to prevent autointoxication through sequestration (Arbuckle et al., 2017). We show that one of these proteins, Sxph, is able not only to protect Na_V_s from toxin poisoning but also to reverse the action of a high-affinity toxin, STX, on frog Na_V_s (Fig. 8), a result that underscores the potential for such toxin sponge proteins to act as agents of toxin resistance. Characterizing how such toxin-binding proteins protect hosts from autointoxication, alone or together with specialized toxin transport pathways, should provide new insights into the fundamental mechanisms of toxin autoresistance and expand understanding of how organisms handle a range of chemical insults and may lead to the discovery of antidotes against various toxic agents.

## Supplementary Material

Table S1 lists Na_V_ inactivation parameters.

Table S2 lists human → poison frog Na_V_1.4 amino acid variants.

Table S3 lists the recovery time from anesthesia (in minutes).

Data S1 provides gene assembly scripts.

## Data Availability

Sequences of *Pum* Na_V_1.4 (GenBank accession no. MZ545383), *Pum* Na_V_1.5 (GenBank accession no. MZ545384), *Pum* Na_V_β2 (GenBank accession no. MZ545385), *Pt* Na_V_1.4 (GenBank accession no. MZ545381), and *Dt* Na_V_1.4 (GenBank accession no. MZ545382) are available from the National Center for Biotechnology Information. Requests for material should be sent to D.L. Minor.
