# Supplementary material for: Evidence that toxin resistance in poison birds and frogs is not rooted in sodium channel mutations and may rely on “toxin sponge” proteins
Source: J Gen Physiol. 2021 Aug 5;153(9):e202112872. doi: 10.1085/jgp.202112872 (PMC8348241; doi:10.1085/jgp.202112872)
Supplement: Table S1 — lists NaV inactivation parameters. [file JGP_202112872_TableS1.docx]

**Table S1** **Na_V_ Inactivation parameters**

|  | Channel | | V_1/2_ _inact_ | ΔV_1/2_ _inact_ | | k_inact_ | n | |
| --- | --- | --- | --- | --- | --- | --- | --- | --- |
| ***Bird*** | ***Pum* Na_V_1.4** | -64.2 ±1.3 | | n.a. | 4.8 ±0.1 | | 14 |  |
|  | ***Pum* Na_V_1.4 + *Pk*Na_V_β2** | -66.5 ±1.4 | | n.a. | 4.6 ±0.2 | | 8 |  |
|  | ***Pum* Na_V_1.5** | -75.9 ±1.6 | | n.a. | 4.2 ±0.5 | | 6 |  |
|  | ***Pum* Na_V_1.4 N432T (DI)** | -61.2 ±1.6 | | n.a. | 9.8 ±0.5 | | 6 |  |
|  | ***Pum* Na_V_1.4 N830T (DII)** | -75.0 ±0.9 | | -10.8 ±1.6 | 4.2 ±0.2 | | 7 |  |
|  | ***Pum* Na_V_1.4 N1306T (DIII)** | -65.3 ±1.0 | | n.a. | 5.1 ±0.5 | | 7 |  |
|  | ***Pum* Na_V_1.4 N1609T (DIV)** | -74.5 ±1.7 | | -10.3 ±2.1 | 4.9 ±0.1 | | 3 |  |
|  | ***Pum* Na_V_1.4 N1609A (DIV)** | -77.6 ±0.9 | | -13.4 ±1.6 | 4.7 ±0.2 | | 10 |  |
| ***Human*** |  |  | |  |  | |  |  |
|  | ***Hs* Na_V_1.4** | -60.5 ±0.8 | | n.a. | 4.7 ±0.3 | | 12 |  |
|  | ***Hs* Na_V_1.4 N1591T (DIV)** | -70.3 ±0.9 | | -9.8 ±1.2 | 4.2 ±0.2 | | 3 |  |
|  |  |  | |  |  | |  |  |
| ***Rat*** | ***Rn* Na_V_1.4** | -61.9 ±0.2 | | n.a. | 6.1 ±0.2 | | 4 |  |
|  | ***Rn* Na_V_1.4 N1584T (DIV)** | -88.9 ±0.4 | | -27.0 ±0.4 | 12.6 ±0.4 | | 6 |  |
| ***Poison frog*** |  |  | |  |  | |  |  |
|  | ***Pt* Na_V_1.4** | -73.9 ±0.6 | | n.a. | 5.9 ±1.4 | | 3 |  |
|  | ***Pt* Na_V_1.4 (N1600T) (DIV)** | -81.6 ±0.7 | | -7.7 ±0.9 | 4.7 ±0.3 | | 6 |  |
|  | ***Dt* Na_V_1.4** | -62.2 ±1.3 | | n.a. | 5.6 ±0.5 | | 8 |  |
|  | ***Dt* Na_V_1.4 (N1600T) (DIV)** | -72.0 ±1.4 | | -9.8 ±1.9 | 5.2 ±0.3 | | 3 |  |

V_1/2_ _inact_ = half-inactivation potential

ΔV_1/2_ _inact =_ V_1/2, inact mutant_ -V_1/2 , inact WT_

k_inact_ = slope factor of inactivation

n= number of independent measurements
