# Supplementary material for: Evidence that toxin resistance in poison birds and frogs is not rooted in sodium channel mutations and may rely on “toxin sponge” proteins
Source: J Gen Physiol. 2021 Aug 5;153(9):e202112872. doi: 10.1085/jgp.202112872 (PMC8348241; doi:10.1085/jgp.202112872)
Supplement: Table S2 — lists human → poison frog NaV1.4 amino acid variants. [file JGP_202112872_TableS2.docx]

**Table S2 Human🡪Poison frog Na_V_1.4 amino acid variants**

| **Location (# of substitutions)** | | | **Human🡪poison frog** | | | **Location** | | | **Human🡪poison frog** | |
| --- | --- | --- | --- | --- | --- | --- | --- | --- | --- | --- |
| **N-term cytoplasmic domain (11)** | |  | I41V  E43D  E46V  S51N  D52G  N58S  K85Q  G94S  S101T  P104K  L106M | | | **VSD- II (9)** | **DII-S0 (1)** | | K584Q |  |
|  |  |  |  |  |  |  | **DII S2 (2)** | | T641A  V645F |  |
|  |  |  |  |  |  |  | **DII S2-S3 loop (3)** | | E655Y  Q659V  I663V |  |
|  |  |  |  |  |  |  | **DII S3-S4 loop (1)** | | Q682E |  |
|  |  |  |  |  |  | **PD-II (2)** | **DII-S6 (2)** | | I807V  A830S |  |
| **VSD-I (8)** | **DI S1 (4)** | | | M130F  T138A  S146N  S152A | | **DII – DIII loop (10)** |  | | A855I  F923L  L929Y  T930R  Q932E  T946E  D958T  K961I  A990T  E991L |  |
|  | **DI S2 (1)** | | | L172F | |  |  |  |  |  |
|  | **DI S2-S3 loop (1)** | | | D180S | |  |  |  |  |  |
|  | **DI S3 (1)** | | | L201T | |  |  |  |  |  |
|  | **DI S3-S4 loop (1)** | | | I210V | |  |  |  |  |  |
| **PD- I (21)** | **DI S5 (2)** | | | | S248A  S259A | **VSD- III (5)** | **DIII S0 (1)** | | L1029V/I* |  |
|  |  |  |  |  |  |  | **DIII S1 (1)** | | V1047I |  |
|  |  |  |  |  |  |  | **DIII S2 (1)** | | R1071K |  |
|  | **DI S5-S6 loop (11)** | | | | D319S  A320T  I322L  A346S  G352S  E354T  S368N  Y369F  T371S  S373N  F376Y |  | **DIII S5-S6 loop (2)** | | K1215R  E1257K/N* |  |
|  |  |  |  |  |  | **VSD- IV (15)** | **DIV S0 (4)** | | Q1341V  K1350P  V1359I  T1360S |  |
|  |  |  |  |  |  |  | **DIV S1 (3)** | | T1367F  V1377I  V1381I |  |
|  |  |  |  |  |  |  | **DIV S2 (5)** | | L1389E  Y1395F  G1407T  V1410F  Y1420F |  |
|  | **DI S6 (8)** | | | | S415A  I419V  A431D  A439Q  D441A  K442L  E446K  Q450D |  | **DIV S3 (1)** | | L1435I |  |
|  |  |  |  |  |  |  | **DIV S3-S4 (1)** | | K1448E |  |
|  |  |  |  |  |  | **PD-IV (4)** | **DIV S5-S6 loop (1)** | | L1566I |  |
|  |  |  |  |  |  |  | **DIV S6 (3)** | | S1581A  V1599I  S1618G |  |
|  |  |  |  |  |  | **C-terminal cytoplasmic domain (3)** | | Q1758H  L1649M  E1658D | |  |
| **DI-DII Loop (10)** |  | | | | E463G  K464Q  K495M  K536T  I548V  H560Q  K562T/P*  H572N  I576K  N578D |  |  |  |  |  |

Pore domain (PD) and voltage sensor domain (VSD) are indicated.

Magenta residues are variants shared with (Tarvin *et al*., 2016).

Variations are denoted using the human residue identity, *Pt* Na_V_1.4 number, and poison frog residue identity.

* indicates site where *Pt* Na_V_1.4 and *Dt* Na_V_1.4 differ.
