# Supplementary material for: Evidence that toxin resistance in poison birds and frogs is not rooted in sodium channel mutations and may rely on “toxin sponge” proteins
Source: J Gen Physiol. 2021 Aug 5;153(9):e202112872. doi: 10.1085/jgp.202112872 (PMC8348241; doi:10.1085/jgp.202112872)
Supplement: Table S3 — lists the recovery time from anesthesia (in minutes). [file JGP_202112872_TableS3.docx]

**Table S3 Recovery time from anesthesia (minutes)**

|  | **PBS** | **BTX** | **STX** | **TTX** |
| --- | --- | --- | --- | --- |
| ***X. laevis*** | 29 ± 1 | 15 ±2 | N/A | N/A |
| ***P.leucomystax*** | 169 ± 12 | 70 ± 20 | N/A | 720 ± 30 |
| ***P. terribilis*** | 10 ± 1 | 14 ± 2 | 72 ± 8 | 354 ± 6 |
| ***D. tinctorius*** | 35 ± 4 | 38 ± 3 | 32 ± 14 | 283 ± 57 |
| ***M. aurantiaca*** | 56 ± 11 | 60 ± 10 | 52 ± 3 | 1011 ± 51 |

Values are average ± S.E.M**.**

N=3 for each condition.
