# Supplementary material for: Evidence that toxin resistance in poison birds and frogs is not rooted in sodium channel mutations and may rely on “toxin sponge” proteins
Source: J Gen Physiol. 2021 Aug 5;153(9):e202112872. doi: 10.1085/jgp.202112872 (PMC8348241; doi:10.1085/jgp.202112872)
Supplement: Data S1 — provides gene assembly scripts. [file JGP_202112872_DataS1.docx]

**Gene assembly scripts.**

#!/bin/sh

#this script should search for reads matching sodium channels, create a seed file from MiSeq and HiSeq reads, and start a PRICE assembly of the sodium channel using these seeds

#these steps use blatq to search for reads matching the sodium channel

echo "$@"_starting blatq search to pull seeds for PRICE

blatq -t=dna /data/jdumbacher/Pitohui_Nav/BlastLibs/SCN4A-Corvus_cornix-Genomic.fa /data/jdumbacher/Pitohui_Nextera/Pitohui_HiSeq_data/Final_Fastq/Pitohui157_HiSeq_ALLDATA.trimmed.fastq /data/jdumbacher/Pitohui_Nav/BlastResults/SCN4A-Corvus_cornix_HiSeq_ALLDATA_matches.m8 -out=blast8

blatq -t=dna /data/jdumbacher/Pitohui_Nav/BlastLibs/SCN4A-Corvus_cornix-Genomic.fa /data/jdumbacher/Pitohui_Nextera/Pitohui_MiSeq_data/Final_BBMerged_Trimmed_Files/Pitohui157_MiSeq_ALLDATA_trimmed.fastq /data/jdumbacher/Pitohui_Nav/BlastResults/SCN4A-Corvus_cornix_MiSeq_ALLDATA_matches.m8 -out=blast8

echo "$@" Blatq done

# Step 2 exerpt reads by id:

echo "$@"Now starting to excerpt reads with blatq hits by ID

/data/jdumbacher/bin/excerptByIDs /data/jdumbacher/Pitohui_Nav/BlastResults/SCN4A-Corvus_cornix_HiSeq_ALLDATA_matches.m8 /data/jdumbacher/Pitohui_Nextera/Pitohui_HiSeq_data/Final_Fastq/Pitohui157_HiSeq_ALLDATA.trimmed.fastq > /data/jdumbacher/Pitohui_Nav/BlastResults/SCN4A-Corvus_cornix_HiSeq_ALLDATA_matches.fastq

/data/jdumbacher/bin/excerptByIDs /data/jdumbacher/Pitohui_Nav/BlastResults/SCN4A-Corvus_cornix_MiSeq_ALLDATA_matches.m8 /data/jdumbacher/Pitohui_Nextera/Pitohui_MiSeq_data/Final_BBMerged_Trimmed_Files/Pitohui157_MiSeq_ALLDATA_trimmed.fastq > /data/jdumbacher/Pitohui_Nav/BlastResults/SCN4A-Corvus_cornix_MiSeq_ALLDATA_matches.fastq

echo "$@"done excerpting reads

cat /data/jdumbacher/Pitohui_Nav/BlastResults/SCN4A-Corvus_cornix_HiSeq_ALLDATA_matches.fastq /data/jdumbacher/Pitohui_Nav/BlastResults/SCN4A-Corvus_cornix_MiSeq_ALLDATA_matches.fastq > /data/jdumbacher/Pitohui_Nav/BlastResults/SCN4A-Corvus_cornix.seeds.fastq

echo "$@"number of seeds in seed file, /data/jdumbacher/Pitohui_Nav/BlastResults/SCN4A-Corvus_cornix.seeds.fastq

grep -c "@" /data/jdumbacher/Pitohui_Nav/BlastResults/SCN4A-Corvus_cornix.seeds.fastq

# for larger read sets, assemble initial reads using spades:

python /data/jdumbacher/SPAdes-3.9.0-Linux/bin/spades.py --careful -s /data/jdumbacher/Pitohui_Nav/BlastResults/SCN4A-Corvus_cornix.seeds.fastq -o /data/jdumbacher/Pitohui_Nav/BlastResults/SCN4A-Corvus_cornix.assembled-seeds

# Assemble using PRICE (For PRICE assemblies - use note for PRICE assemblies Labnotes 25 November 2014)

echo "$@"Now starting PRICE assembly using reads

PriceTI -fp /data/jdumbacher/Pitohui_Nextera/Pitohui_MiSeq_data/Final_BBMerged_Trimmed_Files/Pitohui157_Run1_BBUnmerged_trimmed_paired_R1.fastq /data/jdumbacher/Pitohui_Nextera/Pitohui_MiSeq_data/Final_BBMerged_Trimmed_Files/Pitohui157_Run1_BBUnmerged_trimmed_paired_R2.fastq 549 -fp /data/jdumbacher/Pitohui_Nextera/Pitohui_HiSeq_data/Final_Fastq/Pitohui157_HiSeq_PE_trimmed_R1.fastq /data/jdumbacher/Pitohui_Nextera/Pitohui_HiSeq_data/Final_Fastq/Pitohui157_HiSeq_PE_trimmed_R2.fastq 482 -icf /data/jdumbacher/Pitohui_Nav/BlastResults/SCN4A-Corvus_cornix.assembled-seeds/contigs.fasta 1 1 5 -mol 30 -mpi 90 -MPI 85 -nc 50 -a 10 -target 85 1 1 1 -o /data/jdumbacher/Pitohui_Nav/PriceAssemblies/Pitohui_SCN4A-from_Corvus_cornix.Ass-seeds.fa -o /data/jdumbacher/Pitohui_Nav/PriceAssemblies/Pitohui_SCN4A-from_Corvus_cornix.ass-seeds.priceq -maxHp 25 -logf /data/jdumbacher/Pitohui_Nav/PriceAssemblies/Pitohui_SCN4A-from_Corvus_cornix.ass-seeds.log 2> /data/jdumbacher/Pitohui_Nav/PriceAssemblies/Pitohui_SCN4A-from_Corvus_cornix.ass-seeds.errorlog
